# Mitochondria-targeted metformin analogs activate the ER stress-unfolded protein response pathway to drive apoptosis in pancreatic cancer

**DOI:** 10.1038/s41419-026-08859-y

**Published:** 2026-05-22

**Authors:** Maria Poimenidou, Jordan M. Bobek, Donovan Drouillard, Tyler Harris, Elisabeth Solis, Chad Darnell, Donna McAllister, Robert F. Keyes, Mayumi Ishihara-Aoki, Kazuhiro Aoki, Daisy Sahoo, Balaraman Kalyanaraman, Brian C. Smith, Michael B. Dwinell

**Affiliations:** 1 Department of Microbiology & Immunology, Medical College of Wisconsin, Milwaukee, WI 53226 USA; 2 Center for Immunology, Medical College of Wisconsin, Milwaukee, WI 53226 USA; 3 Department of Biochemistry, Medical College of Wisconsin, Milwaukee, WI 53226 USA; 4 Program in Chemical Biology, Medical College of Wisconsin, Milwaukee, WI 53226 USA; 5 Cancer Center, Medical College of Wisconsin, Milwaukee, WI 53226 USA; 6 Department of Cell Biology, Neurobiology, and Anatomy, Medical College of Wisconsin, Milwaukee, WI 53226 USA; 7 Department of Medicine, Medical College of Wisconsin, Milwaukee, WI 53226 USA; 8 Department of Biophysics, Medical College of Wisconsin, Milwaukee, WI 53226 USA; 9 Department of Surgery, Medical College of Wisconsin, Milwaukee, WI 53226 USA

**Keywords:** Tumour heterogeneity, Apoptosis

## Abstract

Accumulating evidence indicates that evasion of apoptosis and metabolic reprogramming are necessary for pancreatic cancer growth, early invasion, and chemotherapeutic resistance. Building on our prior work, we investigated the anti-tumor potential of a rationally designed mitochondria-targeted variant of the anti-diabetic drug metformin, Mito-Met_10_, in cell culture models and orthotopic xenografts. Using MALDI-mass spectrometry imaging, therapeutic concentrations of fluorinated Mito-Met_10_ were shown to preferentially localize within pancreatic tumors relative to adjacent tissue and liver. Treatment suppressed tumor growth, reduced tumor weights, and increased apoptosis in vivo. Murine and human pancreatic cancer cells demonstrated potent anti-proliferative activity, with low micromolar IC_50_ values, and a concomitant induction of apoptotic programmed cell death in vitro. Seahorse metabolic flux analysis revealed reduced basal and ATP-linked mitochondrial respiration following Mito-Met_10_ treatment without a compensatory increase in glycolysis. Unbiased bulk RNA sequencing revealed significant enrichment of endoplasmic reticulum stress and unfolded protein response pathways, validated by qPCR across pancreatic cancer models, with broad upregulation of UPR-associated genes, establishing that Mito-Met_10_ activation of this stress response is conserved. Mito-Met_10_ caused extensive cytoplasmic vacuolization, mitochondrial swelling, and loss of mitochondrial membrane potential, indicative of severe organelle damage. Mito-Met_10_ activated PERK-eIF2α-ATF4-CHOP signaling, including upregulation of ATF4 and downstream pro-apoptotic transcriptional programs. Pharmacologic inhibition of ISR abrogated apoptotic signaling, demonstrating that PERK-eIF2α-ATF4-CHOP-mediated ISR activation functionally contributes to the anti-tumor effects of Mito-Met_10_. Consistent with these findings, orthotopic tumors from Mito-Met_10_-treated mice exhibited increased nuclear ATF4 staining compared with vehicle controls. Collectively, this study links mitochondrial stress to ER stress-associated apoptosis and identifies mitochondrial stress as a tractable vulnerability that can be manipulated to positively engage anti-tumor responses in pancreatic cancer.

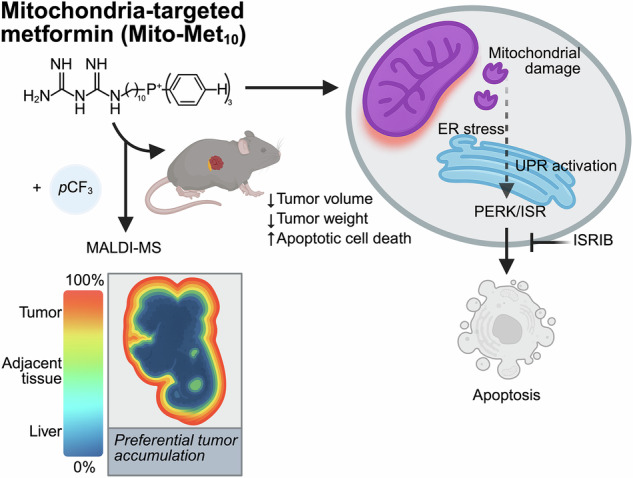

## Introduction

Pancreatic ductal adenocarcinoma (PDAC) is one of the most lethal malignancies, with a five-year survival rate near 10% and limited effective therapeutic options [1]. One of the defining hallmarks of cancer is resistance to apoptosis, which enables tumor cells to survive and proliferate despite genotoxic stress, immune surveillance, or therapeutic intervention [2]. PDAC is no exception, as it employs multiple strategies to suppress cell death, including dysregulation of BCL-2 family proteins, inhibition of caspase activation, and adaptation to metabolic and oxidative stress [3]. Organelle stress, particularly within the endoplasmic reticulum (ER) and mitochondria, plays a pivotal role in tumor cell adaptation and survival. Due to hypoxia, nutrient deprivation, oxidative stress, mitochondrial dysfunction, and high protein synthesis demand, ER stress levels are elevated in solid tumors and drive pro-survival signaling and angiogenesis [4, 5]. These stressors disrupt ER proteostasis, leading to the accumulation of misfolded proteins and activation of the unfolded protein response (UPR). The UPR is coordinated by three ER-resident sensors that attenuate protein translation and engage ER-associated degradation (ERAD) pathways to restore homeostasis [6]. While these adaptive responses promote survival, persistent ER stress can trigger apoptosis, particularly through the PERK-eIF2α-ATF4-CHOP pathway [7, 8]. Elevated expression of ER stress markers, including GRP78/BiP, has been associated with poor prognosis in PDAC [9–13], underscoring the importance of this pathway as both an adaptive mechanism and a therapeutic vulnerability. As such, several experimental studies have examined the UPR and cellular stress as a possible therapeutic target in cancer [14]. Given the severe hypoxia and cell stress present in pancreatic tumors, we hypothesized that further elevation of ER stress in transformed cancer cells may promote apoptosis in PDAC cells [15].

Our research group has harnessed the utility of triphenylphosphonium (TPP^+^) cations to selectively target mitochondrial respiration. Conjugation of TPP^+^ to bioactive molecules has enabled investigations into the role of OXPHOS metabolism on cancer cells [16, 17]. Metformin, a weakly cationic biguanide compound used clinically for type II diabetes, exerts modest inhibitory effects on mitochondrial complex I [18, 19]. However, its clinical application as an anticancer agent has demonstrated limited efficacy in solid and liquid malignancies [20]. Our team rationally synthesized a TPP^+^-conjugated form of metformin, termed Mito-Met_10_ or alternatively MMe, to preferentially accumulate in cells with highly polarized mitochondrial membranes [21]. In both human and murine tumor cells, Mito-Met_10_ exhibited superior potency over metformin in inhibiting complex I, increasing mitochondrial reactive oxygen species, and activating AMP-activated protein kinase (AMPK) [22–24]. Indeed, Mito-Met_10_ was 100–1000-times more potent than metformin in inhibiting tumor cell proliferation in culture and in preclinical xenograft models [17]. While Mito-Met_10_’s anti-tumor effects in vivo were correlated with cell cycle disruption, its precise molecular mechanism of action remains unclear [17, 21–27]. Given the metabolic flexibility of PDAC cells, we suspected that bioenergetic disruption alone may not fully account for the effects of Mito-Met_10_. Mitochondrial stress is increasingly recognized to engage broader stress-adaptive signaling networks beyond metabolic limitation, including ER stress and integrated stress response (ISR) pathways [28]. Given the inter-relationship of the cell cycle and ER stress, we hypothesized that Mito-Met_10_ exerts antitumor activity in part by promoting apoptosis through further elevation and activation of ER stress and UPR signaling. While UPR activation typically promotes tumor survival under conditions of mild or transient stress, excessive or prolonged ER stress may represent an Achilles’ heel that shifts this adaptive pathway toward apoptosis, particularly through activation of the PERK-eIF2α-ATF4-CHOP axis and downstream pro-apoptotic transcriptional programs.

Building on the established roles of mitochondrial dysfunction and ER stress in PDAC, our study investigated the impact of Mito-Met_10_ on ER stress pathways and pro-apoptotic signaling in PDAC cells. Using Seahorse metabolic flux analysis, molecular interrogation of ATF4 signaling, and pharmacologic inhibition of the ISR, we define a mechanistic link between mitochondrial dysfunction, PERK-eIF2α-ATF4-CHOP signaling, and apoptotic cell death. Importantly, using our previously characterized fluorinated Mito-Met_10_ analog [22], we demonstrated preferential intra-tumoral accumulation of Mito-Met_10_, supporting the notion that TPP^+^ functionally enhances tumor-targeting in vivo. Increased localization of Mito-Met_10_ was correlated with increased tumor cell apoptosis and enhanced ATF4 signaling in vivo, confirming Mito-Met_10_ functionally overcomes PDAC resistance to apoptosis. This work advances the understanding of how mitochondrial-selective bioenergetic inhibitors intersect with ER stress and UPR signaling to induce apoptosis, offering a broader framework for exploiting stress-response vulnerabilities across multiple tumor types.

## Results

### Mito-Met_10_ accumulates in PDAC tumors and suppresses tumor growth in vivo

**Fig. 1:**
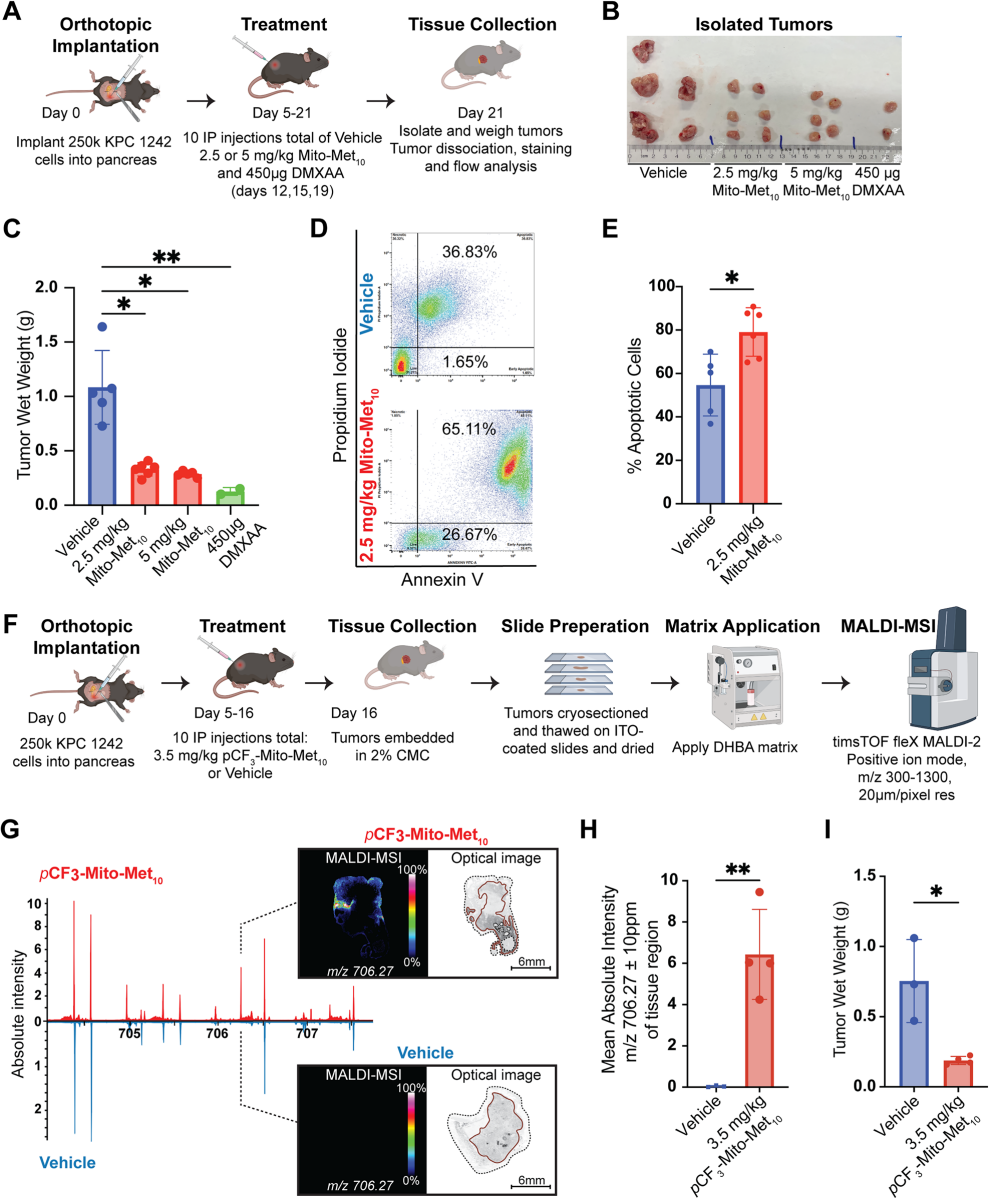
In vivo localization and antitumor activity of Mito-Met_10_. **A** Experimental schematic for assessment of antitumor efficacy. Mice bearing orthotopic KPC 1242 tumors were treated intraperitoneally with either vehicle (*n* = 6; one mouse died during the study), 2.5 mg/kg Mito-Met_10_ (*n* = 6), 5 mg/kg Mito-Met_10_ (*n* = 5; one mouse died during the study), or the STING agonist DMXAA (450 μg/mouse, *n* = 2) as a positive control. **B** Representative images of excised tumors from each treatment group. **C** Tumor weights (g) corresponding to the tumors shown in panel B. Statistical analysis was performed using one-way ANOVA with Dunnett’s multiple comparisons test. **D** In a separate cohort, mice were treated with either vehicle (*n* = 6; one mouse died during the study) or 2.5 mg/kg Mito-Met_10_ (*n* = 6). Representative flow cytometry gating for apoptotic cells is shown. **E** Quantification of Annexin V^+^ apoptotic cells for vehicle and 2.5 mg/kg Mito-Met_10_-treated tumors. Tumors were harvested, enzymatically dissociated into single-cell suspensions, and stained with Annexin V and PI for flow cytometry. Statistical analysis was performed using an unpaired *t*-test. **F** Experimental schematic for quantification of *p*CF3-Mito-Met_10_ in orthotopic pancreatic tumors. C57BL/6 mice were orthotopically implanted with KPC 1242 cells and treated intraperitoneally with vehicle or *p*CF_3_-Mito-Met_10_ (3.5 mg/kg, *n* = 4) for 10 total injections. Tumors were harvested and analyzed by timsTOF fleX MALDI-2 in positive ion mode (m/z 300–1300; 20 μm spatial resolution). **G** Representative mean mass spectra and MALDI-MS ion images showing the fluoride signal at *m/z* 706.27, corresponding to the phosphonium cation of *p*CF_3_-Mito-Met_10_, with matched optical images from *p*CF_3_-Mito-Met_10_-treated (+) and vehicle-treated (−) tumors. Tumor regions are outlined with dashed black lines, with necrotic regions outlined with solid brown lines. Ion distribution maps demonstrate preferential localization of *p*CF_3_-Mito-Met_10_ within pancreatic tumor tissue. **H** Quantification of the mean absolute intensity at m/z 706.27 ±10ppm in pancreas tumor tissues from vehicle (*n* = 3) and *p*CF_3_-Mito-Met_10_-treated (*n* = 4) mice. **I** Tumor weights (g) corresponding to the study in (**F**–**H**). Statistical analysis was performed using an unpaired *t*-test. Values are mean ±SD. *p < 0.05, **p < 0.01, ***p < 0.001, ****p < 0.0001.

To assess therapeutic efficacy, tumor-bearing mice were treated with vehicle, DMXAA, a STING agonist we had previously shown to inhibit murine pancreatic tumor growth in vivo [29, 30], 2.5 mg/kg Mito-Met_10_, or 5 mg/kg Mito-Met_10_ (Fig. 1A). Both Mito-Met_10_ doses significantly reduced tumor weight compared to vehicle-treated mice, with reductions comparable to the STING agonist DMXAA used as a positive control (Fig. 1B, C). To determine whether these effects were associated with apoptosis, tumors from vehicle and 2.5 mg/kg Mito-Met_10_-treatment groups were dissociated into single-cell suspensions and stained with Annexin V and propidium iodide (PI) for flow cytometric quantification. Mito-Met_10_ significantly increased the proportion of Annexin V-positive apoptotic cells relative to vehicle controls (Fig. 1D, E).

A major limitation of our prior studies has been the lack of direct evidence confirming the accumulation of Mito-Met_10_ within tumors. To address this, we employed a preclinical PDAC engraftment model using a fluorinated triphenylphosphonium analog, *p*CF_3_-Mito-Met_10_ (Supplementary Fig. 1), which retains biological activity while enabling in vivo detection by MALDI imaging mass spectrometry using a Bruker timsTOF fleX instrument [22, 31–33]. Prior to tissue imaging, *p*CF_3_-Mito-Met_10_ was analyzed on a conventional stainless steel MALDI plate, confirming robust ionization and detection of the phosphonium cation [M]+ at m/z 706.27, along with minor in-source fragments (Supplementary Fig. 2A, B). Fresh-frozen tissue samples were sectioned at 10 µm thickness and thaw-mounted onto indium tin oxide (ITO)-coated glass slides for imaging mass spectrometry analysis using a timsTOF fleX MALDI instrument.

KPC 1242 cells were orthotopically implanted into the pancreas of C57BL/6 male mice, followed by 10 intraperitoneal injections of vehicle or 3.5 mg/kg *p*CF_3_-Mito-Met_10_ (Fig. 1F). As a control for sensitivity of detection, KPC 1242 cells were heterotopically engrafted to the flank of C57BL/6 mice and injected intratumorally with *p*CF3-Mito-Met_10_(Supplementary Fig. 2C, D). Optical images and corresponding ion distribution map for m/z 706.27 revealed pronounced signal in tumors from the *p*CF_3_-Mito-Met_10_-treated mice compared to vehicle where signal was undetectable (Fig. 1G; Supplementary Fig. 3). Quantitative analysis confirmed a significant increase in mean absolute intensity at m/z 706.27 in tumors from *p*CF_3_-Mito-Met_10_-treated mice compared to vehicle (Fig. 1H). Tumor wet weights measured at study end confirmed that *p*CF_3_-Mito-Met_10_ significantly reduced tumor growth in vivo (Fig. 1I). Minimal localization was detected in the liver (Supplementary Fig. 3), indicating preferential tumor accumulation. Collectively, these findings demonstrate that Mito-Met_10_ preferentially accumulated in PDAC tumors in vivo, reduced tumor burden, and stimulated robust apoptotic responses in tumors.

### Mito-Met_10_ suppresses cell growth and induces apoptotic signaling in PDAC cells

**Fig. 2:**
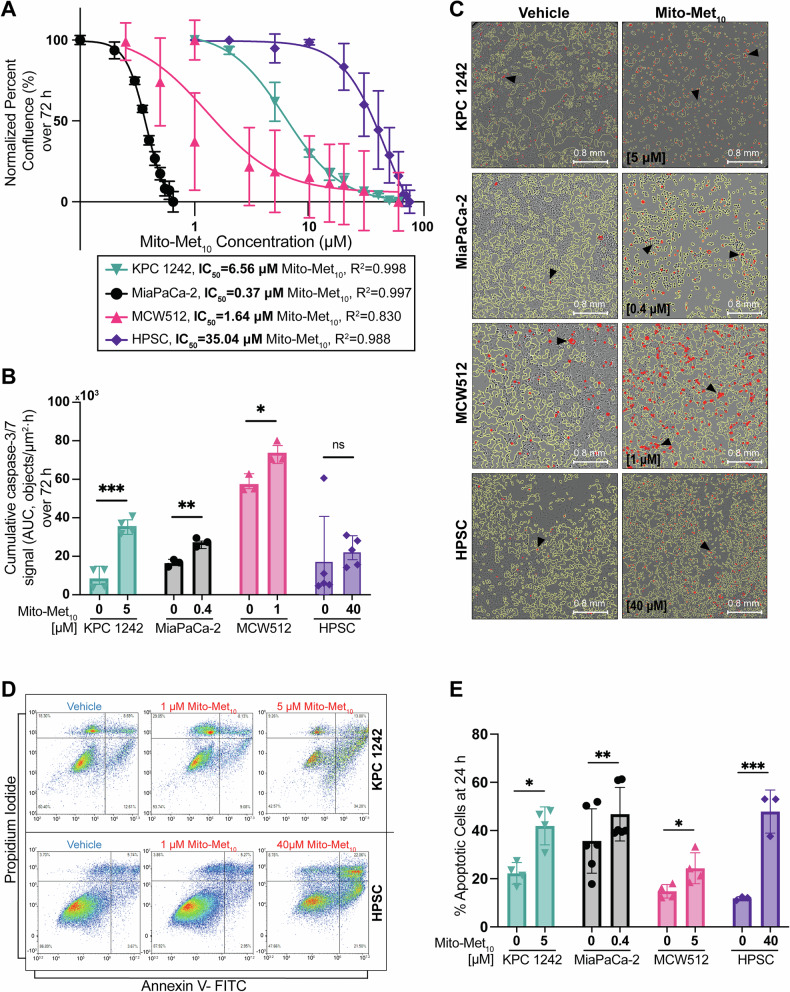
Mito-Met_10_ induces growth inhibition and apoptotic signaling in PDAC cell lines. **A** Kinetic proliferation curves for KPC 1242, MiaPaCa-2, MCW512, and HPSC cells treated with increasing concentrations of Mito-Met_10_. Cell confluence was quantified by IncuCyte live-cell imaging. IC_50_ values at 72 h were determined by nonlinear regression using a four-parameter logistic model (log[inhibitor] vs. normalized response, variable slope) in GraphPad Prism. Data are plotted on a log_10_-scaled x-axis. Kinetic traces represent mean values; each condition includes at least 3 biological replicates. **B** Cumulative caspase-3/7 activity over 72 h following treatment with vehicle or Mito-Met_10_. Caspase-3/7 activity was measured longitudinally and quantified as total green object integrated intensity (μm^2^/image). For each biological replicate activity was summarized as area under the curve (AUC). Vehicle and treatment conditions were compared within each cell line using an unpaired *t*-test. **C** Representative live-cell images of vehicle (left) and Mito-Met_10_-treated (right) cells at 48 h for the doses indicated in (**B**). *Yellow* outlines denote IncuCyte-generated confluence masks, and red fluorescence indicates caspase-3/7-positive apoptotic cells. **D** Representative Annexin V-FITC/propidium iodide flow cytometry plots of KPC 1242 (top) and HPSC (bottom) cells treated with increasing concentrations of Mito-Met_10_ for 24 h. **E** Quantification of Annexin V^+^ apoptotic cells 24 h after treatment with vehicle or Mito-Met_10_. Additional apoptosis data are shown in Supplementary Fig. 4. Statistical comparisons were performed within each cell line using repeated-measures one-way ANOVA with Tukey’s multiple comparisons test. Values are mean ±SD. *p < 0.05, **p < 0.01, ***p < 0.001, ****p < 0.0001, ns not significant.

To determine whether the in vivo antitumor effects of Mito-Met_10_ reflected apoptosis at the cellular level, we evaluated the cytotoxic potency of Mito-Met_10_ in PDAC and non-malignant pancreatic ductal epithelial cells using live-cell imaging and flow cytometry. Kinetic proliferation analysis revealed concentration-dependent growth inhibition across multiple PDAC cell lines. Nonlinear regression analysis of normalized confluence values at 72 h indicated IC_50_ values in the low micromolar range for PDAC cells (KPC 1242 IC_50_ = 6.56 μM; MiaPaCa-2 IC_50_ = 0.37 μM; MCW512 IC_50_ = 1.64 μM). In contrast, the non-malignant pancreatic fibroblast-like stromal cell line HPSC exhibited substantially reduced sensitivity to Mito-Met_10_ treatment, with an IC_50_ ~5–95-fold higher than that observed in PDAC cells (HPSC IC_50_ = 35.04 μM) (Fig. 2A). Kinetic proliferation curves confirmed that PDAC cells exhibited a marked reduction in confluence beginning at low 1–10 μM concentrations whereas HPSC cells maintained near-baseline proliferation until concentrations exceeding 30 μM were reached (Supplementary Fig. 4A, B). Lastly, Mito-Met_10_ did not significantly alter cancer cell migration in transwell chemotaxis assays (Supplementary Fig. 5E, F), indicating that its growth inhibitory effects were not attributable to impaired migratory capacity.

To determine whether growth inhibition was associated with apoptotic signaling, caspase-3/7 activation was monitored longitudinally using a live-cell apoptosis reporter. This reagent is cell membrane-permeable and non-toxic, allowing live-cell visualization of apoptotic progression. Caspase activity was summarized for each biological replicate using area-under-the-curve (AUC) analysis over 72 h, revealing robust treatment-evoked apoptotic signaling compared to vehicle controls across all PDAC cell lines at doses corresponding to their respective IC_50_ values (Fig. 2B). In KPC 1242 and MiaPaCa-2 cells, caspase activity increased significantly as early as 12 h post-treatment and remained elevated through 72 h (Supplementary Fig. 4C). Representative brightfield microscopy images confirmed increased nuclear caspase-3/7 fluorescence in Mito-Met_10_ treated cells relative to vehicle controls (Fig. 2C; Supplementary Fig. 4A). Inhibition of caspase activity with the pan-caspase inhibitor z-VAD-fmk did not rescue Mito-Met_10_ induced growth suppression or apoptotic signaling (Supplementary Fig. 5A–D), supporting the notion that caspase inhibition alone was insufficient to restore proliferation.

To further validate apoptotic cell death, we performed flow cytometric analysis of Annexin V and propidium iodide staining. Representative Annexin V/PI flow cytometry plots for KPC 1242 and HPSC following 24 h treatment are shown in Fig. 2D, with the complete gating strategy for all cell lines provided in Supplementary Fig. 6. Quantification of Annexin V-positive apoptotic cells demonstrated a concentration-dependent increase in apoptosis in PDAC cell lines, reaching statistical significance at 5 μM in KPC 1242 cells, 5 μM in MCW512 cells and 0.4 μM in MiaPaCa-2 cells (Fig. 2E**;** Supplementary Fig. 4D, E). In contrast, HPSC cells exhibited no significant increase in apoptosis until concentrations exceeded ~40 μM, a concentration 8-100-fold higher than that required to induce apoptosis in PDAC cells and comparable to the suppression of proliferation (Fig. 2E; Supplementary Fig. 4E). Collectively, these results demonstrate that Mito-Met_10_ suppresses proliferation and promotes apoptotic signaling in PDAC cells while exhibiting reduced cytotoxicity toward non-malignant fibrotic stromal cells. The concordance between real-time caspase activation and Annexin V/PI staining confirms that the antiproliferative effects of Mito-Met_10_ are driven, at least in part, through induction of apoptosis in PDAC cells.

### Mito-Met_10_ inhibits mitochondrial oxidative phosphorylation in PDAC cells

**Fig. 3:**
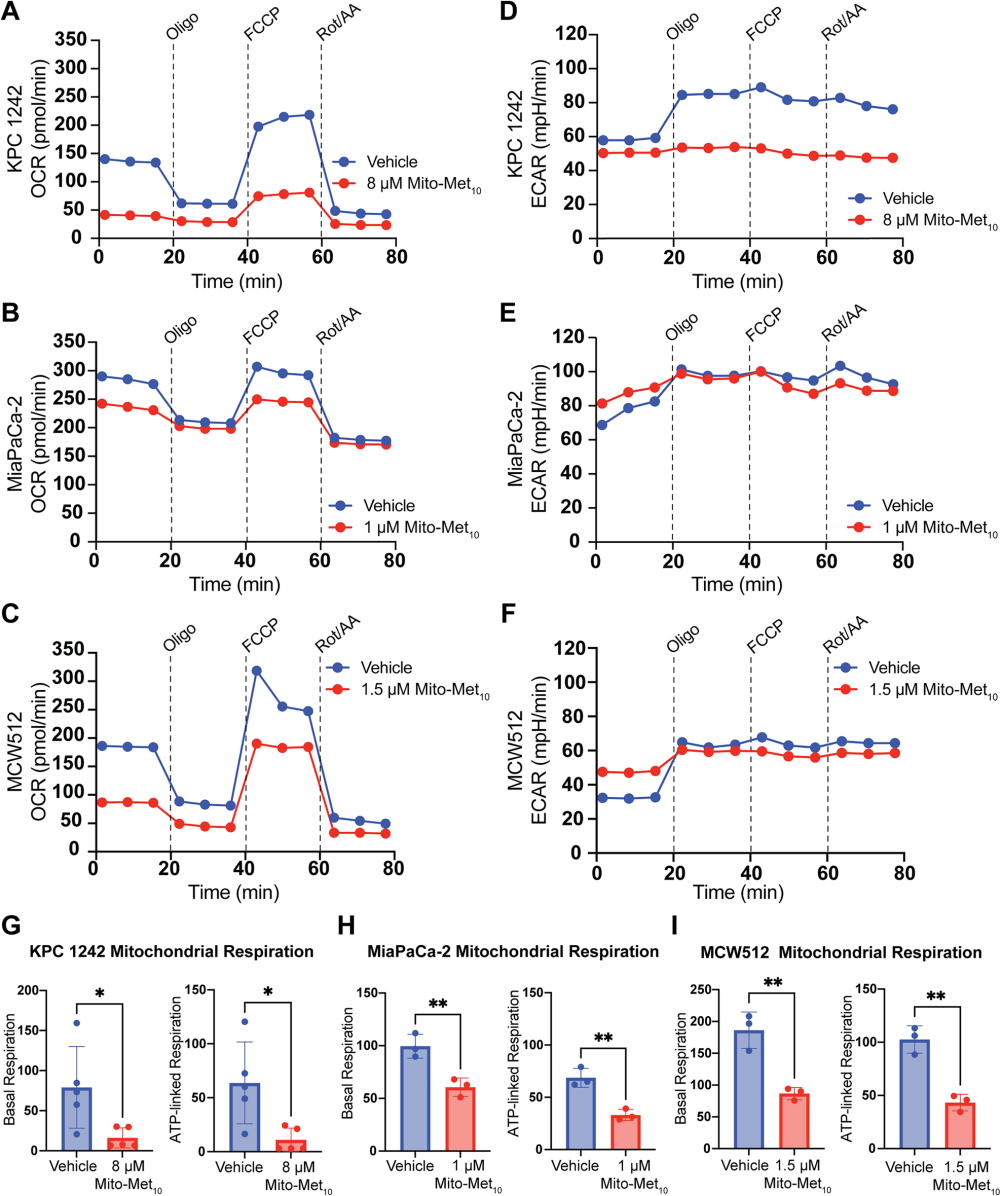
Mito-Met_10_ disrupts mitochondrial respiration in PDAC cell lines as an early response to treatment. Seahorse analysis of oxygen consumption rate (OCR) following 4 h pre-treatment with Mito-Met_10_ in (**A**) KPC 1242 (8 μM), (**B**) MiaPaCa-2 (1 μM) and (**C**) MCW512 (1.5 μM). Mitochondrial function was assessed by sequential injection of oligomycin (1.5 μM), FCCP (1 μM), and rotenone/antimycin A (0.5 μM). Dashed vertical lines indicate injection timing. **D**–**F** Seahorse analysis of extracellular acidification rate (ECAR) following 4 h pre-treatment with Mito-Met_10_ in respective cell lines. **G**–**I** Quantification of basal (left) and ATP-linked (right) mitochondrial respiration calculated from OCR traces in (**A**–**C**). Statistical analysis was performed using an unpaired *t*-test. Additional doses are shown in Supplementary Fig. 7. Values are mean ±SD. *p < 0.05, **p < 0.01, ***p < 0.001, ****p < 0.0001.

Our previous studies demonstrated that Mito-Met_10_ and related mitochondria-targeted metformin analogs inhibit mitochondrial respiration and complex I-derived respiration in pancreatic and colorectal cancer models [17, 22, 23]. Therefore, we next sought to determine whether these effects were associated with impaired mitochondrial bioenergetic function. We performed Seahorse XF mitochondrial stress test analyses in murine (KPC 1242) and human (MiaPaCa-2 and MCW512) PDAC cell lines following exposure to Mito-Met_10_. Real-time metabolic flux analysis revealed that Mito-Met_10_ significantly reduced basal oxygen consumption rate (OCR) and ATP-linked mitochondrial respiration across all PDAC cell lines (Fig. 3A–C, G–I). In contrast, extracellular acidification rate (ECAR) measurements showed no effect following treatment, indicating that inhibition of mitochondrial respiration was not accompanied by a compensatory Warburg-like metabolic shift toward glycolysis (Fig. 3D, E). Further analyses revealed the concentration-dependent reduction in OCR without a change in ECAR (Supplementary Fig. 7). Together, these findings demonstrate that Mito-Met_10_ disrupts respiratory function in PDAC cells without inducing compensatory glycolytic reprogramming. Because mitochondrial respiration supports regeneration of electron acceptors required for metabolite synthesis [34], we also tested whether supplementation with exogenous aspartate or pyruvate could restore growth. Metabolite supplementation produced only modest and transient increases in confluence in KPC 1242 cells and failed to rescue proliferation in MiaPaCa-2 cells (Supplementary Fig. 8), indicating that Mito-Met_10_-induced growth inhibition cannot be explained solely by metabolite limitation.

### Mito-Met_10_ induces mitochondrial structural damage and membrane depolarization

**Fig. 4:**
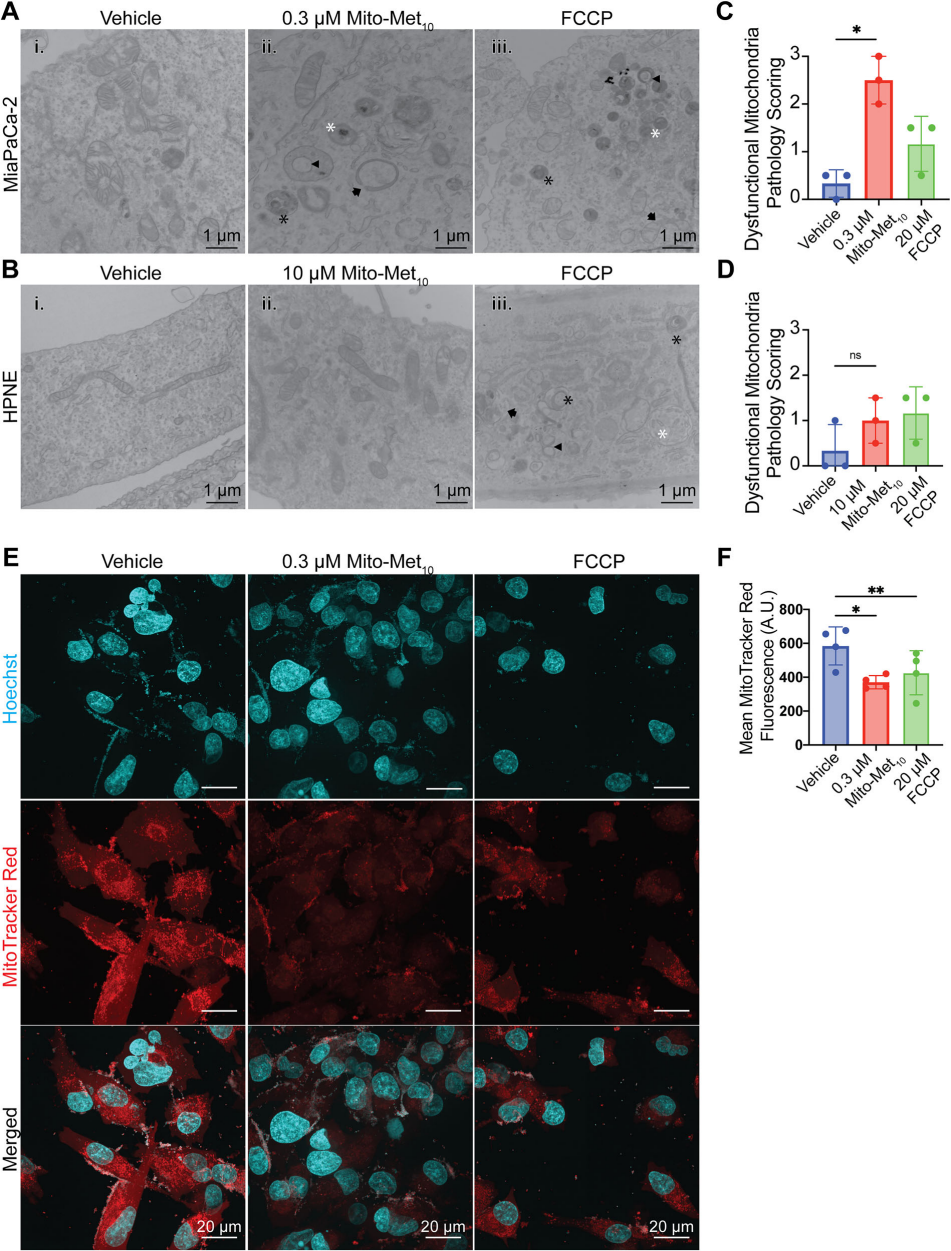
Mito-Met_10_ induces mitochondrial ultrastructural abnormalities in PDAC cells. **A** MiaPaCa-2 and **B** HPNE cells were treated with Mito-Met_10_ or FCCP for 6 h prior to fixation and processing for transmission electron microscopy. Representative images of (i) vehicle, (ii) Mito-Met_10_, and (iii) FCCP (20 μM) treated cells reveal marked mitochondrial ultrastructural abnormalities*. Black arrowheads* indicate vacuolated mitochondria; *black asterisks* denote abnormal mitochondrial morphology, including swelling, disrupted cristae, onion-like concentric membranes; *arrows* highlight mitochondrial branching, and *white asterisks* identify mitochondria undergoing mitophagy. Scale bars, 1 μm. Quantification of mitochondrial pathology scores in (**C**) MiaPaCa-2 and (**D**) HPNE cells. Statistical significance was performed using Kruskal–Wallis test with Dunn’s multiple comparisons. **E** Representative confocal microscopy images of MiaPaCa-2 cells treated with vehicle, Mito-Met_10_ (0.3 μM), or FCCP (20 μM) for 6 h. Cells were stained with Hoechst (nuclei, blue) and MitoTracker Red (mitochondria, red) for 30 min prior to imaging. Merged images are shown in the bottom row. Scale bars, 20 μm. **F** Quantification of Mean MitoTracker Red fluorescence in (**E**) measured from maximum intensity projections of confocal z-stacks in vehicle, Mito-Met_10_, and FCCP-treated cells. Statistical significance was determined using one-way RM ANOVA with Tukey’s multiple comparisons test. Values in (**C**, **D**, **F**) are mean ±SD. *p < 0.05, **p < 0.01, ***p < 0.001, ****p < 0.0001, ns not significant.

Under physiological conditions, mitochondria maintain a highly organized architecture in which the inner boundary membrane folds inward to form tubular or laminar cristae, with crista junctions acting as pores connecting the intermembrane and intracristal spaces [35]. Given that caspase-inhibitors failed to block cell death and Mito-Met_10_ significantly suppressed mitochondrial respiration in PDAC cells, we next sought to determine whether this bioenergetic impairment was accompanied by structural mitochondrial damage and loss of membrane integrity. Transmission electron microscopy (TEM) following 6 h Mito-Met_10_ treatment revealed substantial mitochondrial damage, including swelling, disrupted or lost cristae (*black asterisks*), mitophagy (*white asterisk*), formation of enlarged vacuoles (*black arrowheads)*, and concentric onion-like membrane formations lacking organized cristae (*black asterisks)* (Fig. 4A-ii). Additional ultrastructural features noted in Mito-Met_10_-treated cells and not the control or HPNE-treated cells included extensive branching, blebbing, and inner membrane compartmentalization.

To contextualize the extent of mitochondrial disruption, we compared Mito-Met_10_ with FCCP, a well-established mitochondrial uncoupling agent. Morphologic scoring of mitochondrial morphology demonstrated that Mito-Met_10_ provoked more severe mitochondrial damage than 20 μM FCCP in MiaPaCa-2 cells (Fig. 4A-iii, C). In contrast, HPNE cells, non-transformed pancreatic ductal epithelial cells used as a surrogate for normal exocrine duct cells, displayed only minor mitochondrial changes, even when treated with Mito-Met_10_ at concentrations 30-fold higher than those used in MiaPaCa-2 tumor cells (Fig. 4B-ii, D). Congruent with the mitochondrial ultrastructural abnormalities observed by TEM, confocal imaging with MitoTracker Red revealed significant reductions in mitochondrial fluorescence following Mito-Met_10_ treatment (Fig. 4E, F). Vehicle-treated cells displayed strong mitochondrial signal and an interconnected mitochondrial network, whereas Mito-Met_10_-treated cells exhibited substantially diminished mitochondrial fluorescence intensity. FCCP treatment produced a comparable reduction in signal.

**Fig. 5:**
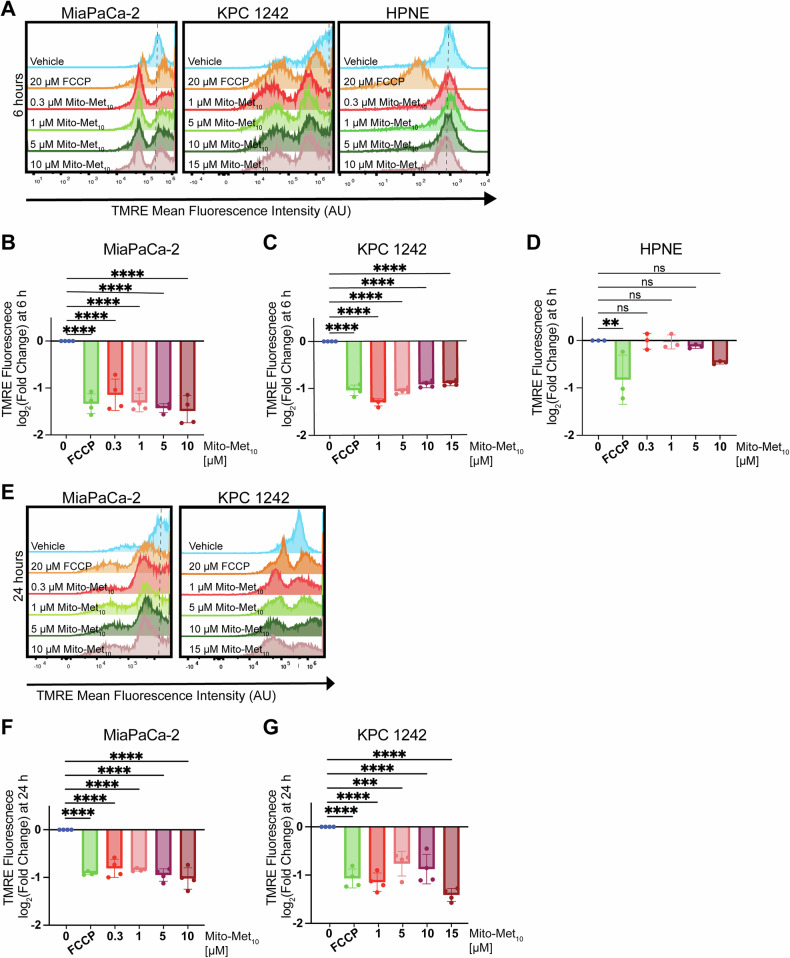
Mitochondrial membrane potential depolarization after Mito-Met_10_ treatment. MiaPaCa-2, KPC 1242, and HPNE cells were treated for (**A**–**D**) 6 h or (**E**–**G**) 24 h with vehicle, as a negative control, 20 μM FCCP, as a depolarization positive control, or increasing concentrations of Mito-Met_10_. Cells were stained with 200 nM TMRE, and mitochondrial membrane potential was measured by flow cytometry. Representative overlaid histograms depict TMRE fluorescence intensity (MFI) under each treatment condition. Quantification of log_2_-transformed fold change in TMRE MFI relative to vehicle control at 6 h for (**B**) MiaPaCa-2, (**C**) KPC 1242, and (**D**) HPNE cells. Quantification of log_2_-transformed fold change in TMRE MFI relative to vehicle control at 24 h for (**F**) MiaPaCa-2 and (**G**) KPC 1242 cells. Statistical analysis was performed using one-way ANOVA with Dunnett’s multiple comparisons test. Values are mean ±SD. *p < 0.05, **p < 0.01, ***p < 0.001, ****p < 0.0001, ns not significant.

Mitochondrial membrane potential was then assessed using tetramethylrhodamine ethyl ester (TMRE), a positively charged membrane-permeable dye. TMRE accumulates within mitochondria in proportion to the mitochondrial membrane potential (ΔΨ_m_), with higher accumulation in metabolically active mitochondria, whereas depolarized, damaged, or dysfunctional mitochondria are unable to sequester TMRE [36]. Mito-Met_10_ treatment produced a dose-dependent decrease in TMRE fluorescence in MiaPaCa-2 and KPC 1242 cells at both 6 h (Fig. 5A–C) and 24 h (Fig. 5E–G), indicating mitochondrial depolarization. FCCP produced similar reductions in TMRE signal, confirming disruption of mitochondrial membrane integrity [23]. In contrast, HPNE cells exhibited minimal TMRE changes (Fig. 5D), consistent with the limited ultrastructural alterations observed by TEM. These results demonstrate that Mito-Met_10_ induces profound structural and functional mitochondrial damage in PDAC cells while largely sparing non-transformed pancreatic epithelial cells at low micromolar concentrations. These mitochondrial morphological hallmarks have been associated with bioenergetic collapse, mitochondrial outer membrane permeabilization, and apoptotic signaling [37–39], supporting a role for mitochondrial damage in Mito-Met_10_-driven apoptosis.

### Bulk transcriptomic analysis of Mito-Met_10_-treated PDAC cells

**Fig. 6:**
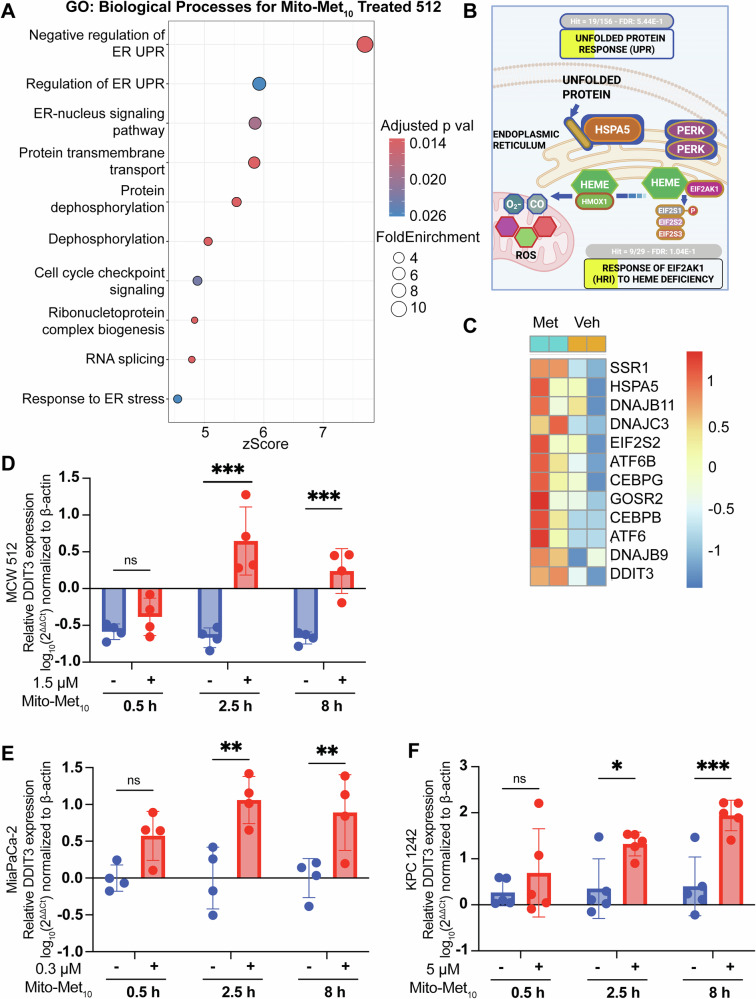
Bulk RNA sequencing reveals activation of ER stress and UPR signaling following Mito-Met_10_ treatment. **A** Gene Ontology (GO) analysis of differentially expressed genes from bulk RNA sequencing of MCW512 cells treated with Mito-Met_10_ (1 μM, 6 h), showing enrichment of ER stress and unfolded protein response (UPR)-related biological processes. Circle size corresponds to fold enrichment, and color indicates adjusted *p*-value. **B** Reactome pathway enrichment analysis (Reactome ID: 37941124) illustrating activation of UPR signaling in Mito-Met_10_-treated MCW512 cells. Diagram generated using BioRender. **C** Heatmap showing upregulation of UPR-related genes in Mito-Met_10_-treated MCW512 cells relative to vehicle controls. Quantitative RT-PCR analysis of *DDIT3* expression in **D** MCW512 (1.5 μM), **E** MiaPaCa-2 (0.3 μM), and **F** KPC 1242 (5 μM) cells treated with Mito-Met_10_ for 0.5, 2.5, or 8 h. Expression levels were normalized to β-actin and presented as log_10_-transformed 2^−ΔΔCt^ relative expression. Statistical analysis was performed using two-way ANOVA with Sidak’s multiple comparisons test. Values are mean ±SD. *p < 0.05, **p < 0.01, ***p < 0.001, ****p < 0.0001, ns not significant.

To define molecular pathways underlying the functional and structural mitochondrial perturbations stimulated by Mito-Met_10_, we performed unbiased bulk RNA sequencing on patient-derived PDAC cells treated with vehicle or Mito-Met_10_. Transcriptomic analysis revealed robust upregulation of ER stress and apoptosis pathways in pancreatic tumor cells following Mito-Met_10_ treatment (Fig. 6A). Gene ontology (GO) analysis identified top-enriched biological processes, including *negative regulation of the ER UPR*, *response to ER stress*, *protein transmembrane transport*, and *protein dephosphorylation*, consistent with a global shift toward proteotoxic stress signaling.

Activation of ER stress and UPR pathways has been linked to inter-organelle stress signaling [40]. In PDAC, dysregulated ER-mitochondria communication contributes to tumor stress adaptation and progression [41]. ER and mitochondria form closely associated contact sites that coordinate calcium, lipid, and metabolite exchange and integrate cellular stress responses, including activation of the UPR [42]. Mitochondrial reactive oxygen species generated at respiratory complexes I and III can further amplify ER stress signaling responses [43–45], while prolonged UPR activation promotes mitochondrial membrane collapse and apoptosis through CHOP-ERO1α-Ca²⁺ dependent pathways [46].

**Fig. 7:**
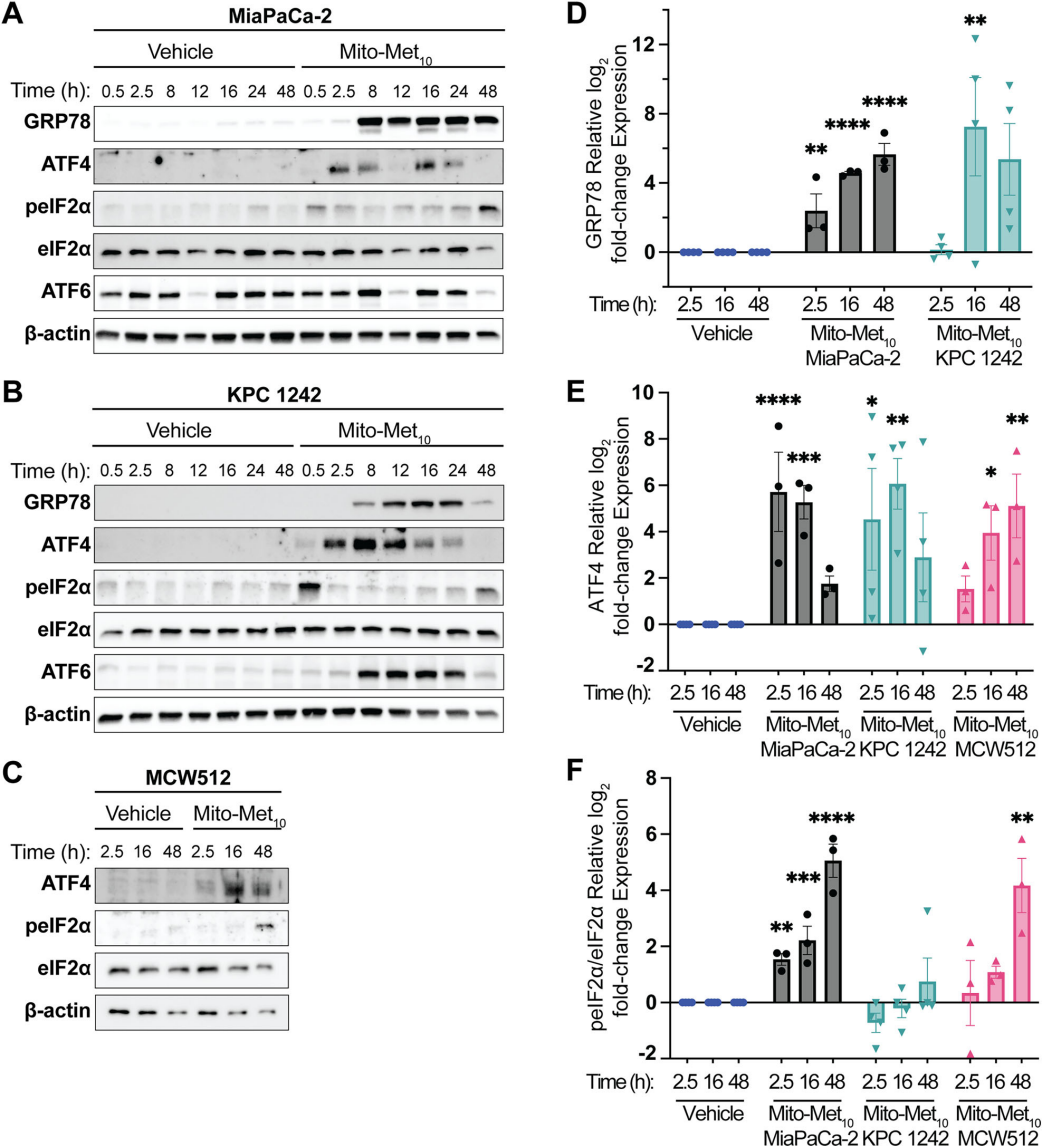
Mito-Met_10_ activates PERK-associated ER stress signaling in human and murine PDAC cells. **A** MiaPaCa-2, **B** KPC 1242, and **C** MCW512 cells were treated with vehicle or Mito-Met_10_ (MiaPaCa-2: 0.3 μM; KPC 1242: 5 μM; MCW512: 1.5 μM) for the indicated time points. Whole-cell lysates were analyzed by immunoblot for GRP78/BiP, ATF4, phosphorylated eIF2α (p-eIF2α), total eIF2α, ATF6, and β-actin as a loading control. Representative blots from at least three independent experiments are shown. Densitometric quantification of **D** GRP78/BiP, **E** ATF4, and **F** p-eIF2α/eIF2α expression normalized to β-actin was performed using Image Lab software. Statistical comparisons were made relative to vehicle controls at the corresponding time point within each cell line using two-way ANOVA with Sidak’s multiple comparisons test. Additional time-point quantification data are shown in Supplementary Fig. 10. Data are presented as mean ±SEM. *p < 0.05, **p < 0.01, ***p < 0.001, ****p < 0.0001.

Pathway enrichment analysis using the Reactome Database (ID: 37941124) further confirmed significant activation of pathways associated with the Unfolded Protein Response, Cellular Response to Mitochondrial Stress, and Cellular Response to Hypoxia (Supplementary Fig. 9). Among these, the *UPR and Response of EIF2AK1 (HRI) to HEME Deficiency* pathways showed the strongest statistical enrichment, suggesting engagement of an integrated stress response involving canonical ER stress sensors and heme-sensing kinases (Fig. 6B). Several differentially expressed genes driving these enrichments included canonical UPR components such as DNA damage-inducible transcript 3 protein DDIT3 (also known as CHOP), ATF6, HSPA5 (BiP), DNAJB9, and EIF2S2, many of which are centrally involved in mediating ER stress-induced apoptosis (Fig. 6C). Quantitative RT-PCR demonstrated significant time-dependent upregulation of DDIT3 in MCW512, MiaPaCa-2, and KPC 1242 cells following Mito-Met_10_ treatment (Fig. 6D–F), with primer sequences provided in Supplementary Table 1. These findings suggested that Mito-Met_10_ activates transcriptional programs linking mitochondrial dysfunction to the unfolded protein response and ER stress-associated apoptosis in pancreatic cancer cells. Given the strong transcriptional enrichment of UPR pathways and the pronounced mitochondrial damage observed by TEM and measured by TMRE, we next assessed whether mitochondrial dysfunction engaged canonical ER stress signaling at the protein level, focusing on the pro-apoptotic PERK-eIF2α-ATF4-CHOP axis (Fig. 7; Supplementary Fig. 10).

### Mito-Met_10_ activates PERK-eIF2α-ATF4-CHOP signaling in PDAC cells

We first examined activation of the adaptive ATF6-BiP arm of the UPR, which promotes chaperone induction and recovery of protein-folding capacity [47, 48], in PDAC cells treated with Mito-Met_10_. In MiaPaCa-2 cells, BiP protein levels increased significantly across all time points (0.5–48 h), while in KPC 1242 cells, BiP expression increased at 16 and 48 h. Notably, ATF6 levels remained unchanged in both cell lines (Fig. 7D; Supplementary Fig. 10G–K). These findings indicate that Mito-Met_10_ induces BiP expression without measurable activation of the ATF6 arm of the UPR, suggesting that PDAC cells fail to mount an effective adaptive UPR response.

Building on the observed apoptosis, mitochondrial dysfunction, and transcriptional evidence of UPR activation, we also examined whether Mito-Met_10_ activates the PERK-eIF2α-ATF4-CHOP signaling axis, the canonical pro-apoptotic branch of the UPR. Immunoblot analysis revealed increased phosphorylation of eIF2α (Ser51) and induction of ATF4 following Mito-Met_10_ treatment in MiaPaCa-2, KPC 1242, and MCW512 cells. In MiaPaCa-2 cells, Mito-Met_10_ induced a robust and sustained increase in p-eIF2α across the treatment time course. This phosphorylation was accompanied by significant ATF4 upregulation beginning at 2.5 h and persisting through 24 h (Fig. 7A, E, F; Supplementary Fig. 10A, D). Further, DDIT3/CHOP expression was also significantly increased beginning at 2.5 h (Fig. 6E). In MCW512, Mito-Met_10_ treatment resulted in a delayed PERK-eIF2α-ATF4 response. Both p-eIF2α and ATF4 increased significantly at later 16–48 h time points (Fig. 7C, E, F; Supplementary Fig. 10C, F), accompanied by increased DDIT3/CHOP expression (Fig. 6D). In KPC 1242 cells, while p-eIF2α was transient, ATF4 expression increased significantly between 2.5 to 24 h (Fig. 7B, E, F; Supplementary Fig. 10B, E). DDIT3 expression was also significantly elevated beginning at 2.5 h with further increases at 8 h (Fig. 6F). For KPC 1242 cells, ATF4 expression may be regulated *via* both eIF2α-dependent and -independent mechanisms, contributing to downstream PERK-associated ER stress signaling. Overall, these data demonstrate that Mito-Met_10_ preferentially engages the pro-apoptotic arm of the UPR, consistent with the apoptotic phenotype observed.

### ER stress signaling contributes to Mito-Met_10_-stimulated apoptosis

**Fig. 8:**
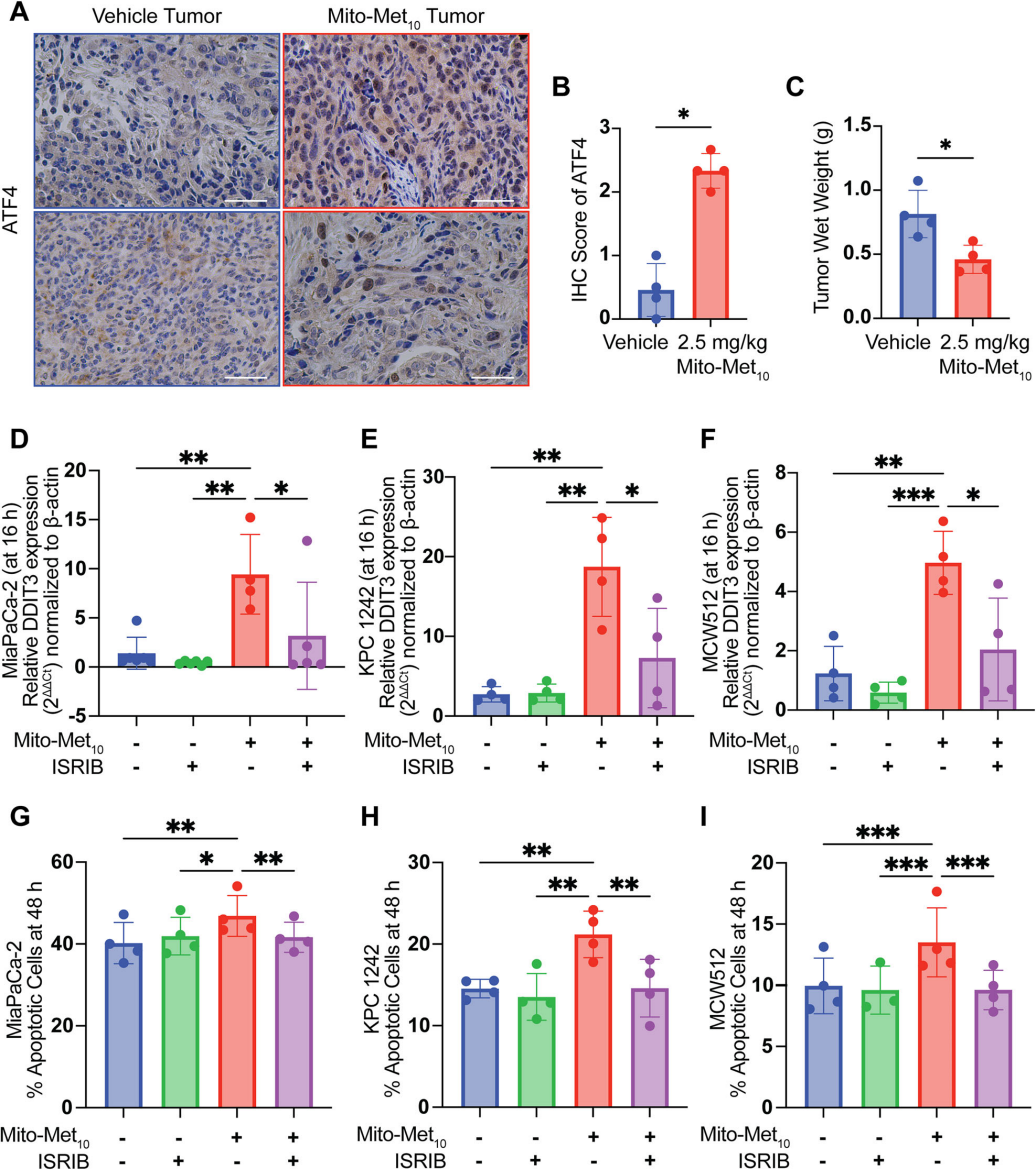
ISR inhibition attenuates Mito-Met_10_-induced stress signaling and apoptosis. **A** Representative immunohistochemical staining for ATF4 in tumor sections from vehicle-treated (*n* = 4) and Mito-Met_10_-treated mice (2.5 mg/kg, *n* = 4) under the same experimental timeline described in Fig. 1A. Scale bars, 20 μm. **B** Quantification of ATF4 IHC scores in tumor samples. ATF4 staining intensity was scored as 0 (negative), 1 (very weak), 2 (moderate), or 3 (strong). Statistical comparison was performed by Mann–Whitney test. **C** Tumor weights (g) corresponding to the study in (**A**, **B**). Quantitative RT-PCR analysis of DDIT3 expression in **D** MiaPaCa-2, **E** KPC 1242, and **F** MCW512 cells treated with vehicle or Mito-Met_10_ in the presence or absence of ISRIB (500 nM). Cells were treated with Mito-Met_10_ at cell line specific concentrations (MiaPaCa-2: 0.4 μM; KPC 1242: 5 μM; MCW512: 1.5 μM) for 16 h. Expression was normalized to β-actin and presented as 2^-ΔΔCT^ relative expression. Statistical comparisons were performed using repeated measures one-way ANOVA with Tukey’s multiple comparisons test. Quantification of Annexin V^+^ apoptotic cells in **G** MiaPaCa-2, **H** KPC 1242, and **I** MCW512 cells treated with vehicle or Mito-Met_10_ in the presence or absence of ISRIB as in (**D**–**F**) for 48 h. Apoptotic cells were defined as Annexin V-positive populations following Annexin V-FITC/propidium iodide staining and flow cytometry analysis. Statistical comparisons were performed using repeated-measures one-way ANOVA with Tukey’s multiple comparisons test. Values are mean ±SD. *p < 0.05, **p < 0.01, ***p < 0.001, ****p < 0.0001.

To determine whether activation of PERK-eIF2α-ATF4-CHOP signaling contributes functionally to Mito-Met_10_-induced apoptosis, we evaluated this axis in orthotopically engrafted PDAC tumors in vivo. Immunohistochemical analysis revealed increased ATF4 in tumors from Mito-Met_10_-treated mice (2.5 mg/kg, *n* = 4) compared with vehicle controls (*n* = 4) (Fig. 8A; Supplementary Fig. 11A). Quantitative scoring confirmed significantly elevated ATF4 staining intensity in treated tumors, with prominent nuclear localization (Fig. 8B), consistent with activation of PERK-eIF2α-ATF4-CHOP signaling in vivo. Wet weights measured at study end confirmed the expected functional inhibition of tumor growth in vivo (Fig. 8C). PERK signals through eIF2α phosphorylation, the convergence point of the broader integrated stress response (ISR). To assess the functional contribution of ISR to Mito-Met_10_-mediated stress responses, PDAC cell lines were treated with Mito-Met_10_ in the presence or absence of the ISR inhibitor ISRIB (500 nM), which stabilizes eIF2B downstream of eIF2α phosphorylation. Pharmacologic inhibition of ISR signaling significantly attenuated DDIT3/CHOP expression in MiaPaCa-2, KPC 1242, and MCW512 cells treated with Mito-Met_10_ (Fig. 8D–F). Concurrently, ISRIB treatment significantly reduced Annexin V-positive apoptotic populations in each of the Mito-Met_10_-treated PDAC cell lines (Fig. 8G–I; Supplementary Fig. 11B–D). Collectively, these data indicate that Mito-Met_10_’s anti-tumor effects functionally reflect PERK-eIF2α-ATF4-CHOP-mediated ISR signaling and apoptotic cell death in vitro and in vivo.

## Discussion

Cellular homeostasis, maintained by the endoplasmic reticulum and coordinated through the UPR, is essential for cell survival under stress. Under mild stress, the UPR promotes adaptation by reducing protein synthesis and increasing chaperone expression; however, under prolonged or severe ER stress, the response shifts toward apoptosis through activation of pro-apoptotic transcription factors such as DDIT3/CHOP [49–51]. Thus, ER stress and UPR signaling represent a double-edged sword; supporting cancer cell survival in adverse environments while providing a potential therapeutic vulnerability when adaptive capacity is exceeded [52, 53]. Understanding and targeting this homeostatic pathway could improve drug delivery and ultimately treatment outcomes [54].

Mitochondria are closely intertwined with ER homeostasis through metabolic and redox coupling. Using cell culture and in vivo murine models, we uncovered a key mechanistic vulnerability connecting mitochondrial respiration and the ER stress response that can be exploited to overcome apoptotic resistance in PDAC. Through a rationally designed, fluorinated small molecule inhibitor of mitochondrial respiration, we observed cancer cell selective accumulation accompanied by pronounced induction of apoptosis in vivo. This compound, Mito-Met_10_, strongly inhibited cell proliferation and oxidative phosphorylation with low IC_50_ values and selectively activated UPR signaling, culminating in apoptosis of human and murine PDAC cells. These data suggest that PDAC chemoresistance may be overcome using mitochondrial-targeted compounds and that these targeted cancer cells retain responsiveness to apoptosis-promoting therapies.

Many cancer cells, including PDAC, harbor hyperpolarized mitochondrial membranes that favor ATP generation but simultaneously sensitize them to triphenylphosphonium (TPP⁺)-conjugated mitochondria-targeted agents [55]. These compounds exploit the hyperpolarized membrane potential to drive selective accumulation within tumor cells. Our prior work demonstrated that such compounds disrupt OXPHOS, elevate mitochondrial reactive oxygen species, and induce profound metabolic stress. Metformin, a widely used antidiabetic drug, exerts anticancer effects by inhibiting mitochondrial complex I; however, its potency is limited [19]. Conjugation of metformin to a TPP^+^ moiety led to the development of mitochondria-targeted analogs such as Mito-Met_10_ (MMe) [17]. These analogs preferentially accumulate in mitochondria, where they inhibit complex I, deplete ATP, and promote ROS generation [16, 17, 19, 21–27]. We previously demonstrated that Mito-Met_10_ was at least 100–1000-fold more potent than metformin in PDAC models, mediating its anticancer effects through AMPK activation, inhibition of mitochondrial complex I-dependent respiration, and stimulation of superoxide and hydrogen peroxide formation. Seahorse metabolic flux analyses revealed that Mito-Met_10_ markedly reduced basal and ATP-linked oxygen consumption across multiple PDAC cell lines, consistent with inhibition of mitochondrial respiration. Importantly, Mito-Met_10_ treatment did not produce an increase in extracellular acidification rate suggesting that mitochondrial inhibition was not accompanied by a strong compensatory shift toward glycolysis. Supporting this interpretation, supplementation with exogenous aspartate or pyruvate produced only modest or transient effects on cell growth, suggesting that the anti-proliferative activity of Mito-Met_10_ cannot be explained solely by electron-acceptor limitation or impaired metabolite regeneration.

Despite prior evidence for Mito-Met_10_’s superior potency over native metformin [17], the precise molecular mechanisms by which it impairs cancer cell proliferation have remained enigmatic. Given PDAC’s remarkable metabolic flexibility, we hypothesized that bioenergetic disruption could not fully explain its anti-tumor effects and proposed that ER stress and UPR activation contribute to its mechanism of action. Our findings confirm that Mito-Met_10_ not only disrupts mitochondrial structure and function but also activates ER stress signaling. We observed robust activation of PERK-eIF2α-ATF4-CHOP signaling, accompanied by BiP induction without measurable activation of the ATF6 arm.

Although UPR and ISR are mechanistically distinct (the UPR encompasses all three sensor branches whereas the ISR broadly converges on eIF2α phosphorylation) PERK functions at their intersection, and the pro-apoptotic output of PERK signaling is appropriately characterized as ISR-dependent, as demonstrated by its sensitivity to ISRIB. The kinetics of PERK-eIF2α-ATF4 activation differed across PDAC cell lines. In MiaPaCa-2 cells, which are more sensitive to Mito-Met_10_, p*-*eIF2α induction occurred rapidly and was sustained, whereas in MCW512 cells, phosphorylation was delayed and became evident only at later time points. KPC 1242 cells displayed significant ATF4 induction without detectable increases in p-eIF2α. Such divergence has been reported in other stress contexts and may reflect differences in metabolic state, kinase engagement, or rapid turnover of phosphorylated eIF2α. Because ATF4 translation can be regulated through multiple ISR kinases and transient phosphorylation events [56], these findings suggest that Mito-Met_10_ engages pro-apoptotic ISR signaling with cell-line-specific kinetics. Upregulation of pro-apoptotic factors such as DDIT3/CHOP further supports the conclusion that prolonged ER stress drives apoptosis in these models. Consistent with this mechanism, pharmacologic inhibition of ISR signaling with ISRIB attenuated DDIT3 induction and reduced Annexin V-positive apoptotic populations across PDAC cell lines, demonstrating that ISR activation functionally contributes to Mito-Met_10_-induced cell death. Evidence of PERK activation was also observed in vivo as orthotopic tumors from Mito-Met_10-_treated mice displayed increased nuclear ATF4 staining relative to vehicle controls. Together, these findings support a model in which mitochondrial dysfunction induced by Mito-Met_10_ triggers PERK-eIF2α-ATF4-CHOP-mediated ISR signaling that culminates in ER stress-mediated apoptosis. Using a fluorinated analog, we further confirmed preferential intratumoral accumulation of Mito-Met_10_, directly linking tumor localization with apoptosis induction of this stress pathway in vivo while observing minimal liver localization. These results underscore the tumor-targeting capacity of TPP⁺ conjugation and provide a mechanistic rationale for exploiting mitochondrial dysfunction to trigger ER stress-associated apoptosis.

Our findings agree with reports of other organelle stress-inducing agents, such as nemorosone and fisetin, which engage both mitochondrial and ER stress pathways to induce apoptosis [57, 58]. PDAC’s high basal ER stress likely creates a therapeutic window in which additional stressors overwhelm adaptive capacity and push cells toward death [9]. Accordingly, our data position Mito-Met_10_ as an ER stress amplifier that deliberately escalates proteostatic burden to overcome PDAC’s resistance to apoptosis. This approach complements other strategies such as inhibition of the hexosamine biosynthetic pathway, which sustains UPR signaling while suppressing EGFR-Akt survival pathways to reverse gemcitabine resistance [59]. Therefore, combining Mito-Met_10_ with context-specific interventions, including established chemotherapeutics like gemcitabine, may provide synergistic benefits and improve clinical outcomes. However, ER stress signaling is highly context-dependent and not uniformly pro-death. Depending on its duration and magnitude, activation of PERK, IRE1, or ATF6 can alternatively promote tumor survival, angiogenesis, or immune evasion [6]. PDAC’s profound metabolic plasticity further complicates therapeutic targeting, as tumors exploit autophagy, macropinocytosis, and stromal nutrient exchange to sustain energy production under stress [60]. Moreover, subclonal heterogeneity adds another layer of complexity; for example, subsets with GCN2-ATF4-driven asparagine synthesis can resist mitochondrial inhibition by maintaining biosynthetic flux, whereas asparaginase restores sensitivity to these inhibitors [61]. Such insights may inform rational combination strategies pairing Mito-Met_10_ with metabolic or nutrient stress modulators.

While our findings consistently showed upregulation of the ER chaperone protein GRP78/BiP following Mito-Met_10_ treatment, elevated GRP78 expression in PDAC has been linked to poor prognosis, cancer stemness, and therapeutic resistance [11]. However, despite robust BiP induction, we did not observe measurable activation of the ATF6 arm of the UPR. BiP upregulation can occur through multiple stress-responsive pathways, including ATF4/CHOP-dependent transcription downstream of PERK or signaling through the IRE1-XBP1 axis, and therefore may not require canonical ATF6 activation. This dissociation may suggest that PDAC cells initiate chaperone induction without engaging a sustained ATF6-mediated adaptive response, potentially rendering them more susceptible to PERK-eIF2α-ATF4-CHOP-mediated apoptosis. In patient tumor samples, GRP78 expression is significantly higher in PDAC tissues than in adjacent normal ducts and correlates with advanced disease stage and poor survival [62]. These findings support the idea that PDAC’s dependence on GRP78-mediated proteostasis may explain its selective vulnerability to Mito-Met_10_, while the lack of ATF6 activation reflects a compromised adaptive response. In contrast, non-malignant pancreatic epithelial cells, which express lower GRP78 levels, are comparatively resistant to Mito-Met_10_.

Studies of Mito-Met_10_ and other mitochondria-targeted agents further highlight opportunities and caveats for exploiting mitochondrial stress as a therapeutic strategy and as a tool to dissect bioenergetic regulation in cancer. Ciclopirox, for example, impairs mitochondrial respiration and induces colorectal cancer cell death *via* PERK-eIF2α-ATF4-CHOP signaling, with the caveat that it also triggers compensatory glycolysis [63], a metabolic adaptation not observed with Mito-Met_10_. Similarly, the clinical-grade complex I inhibitor IACS-010759 induces compensatory glycolysis across multiple cancer models, limiting its single-agent efficacy [64, 65]. Combining IACS-010759 with glycolytic inhibitors such as 2-deoxyglucose significantly enhanced apoptosis [66], highlighting the therapeutic potential of dual metabolic targeting to prevent adaptive bioenergetic switching. IACS-010759 has also been shown to increase mitochondrial fission and mitophagy in acute myeloid leukemia models through AMPK activation and mTOR suppression [67], consistent with the mitochondrial stress responses observed in PDAC cells treated with Mito-Met_10_. Although our study did not directly assess mitophagy, these findings suggest that combining Mito-Met_10_ with autophagy inhibitors may further augment apoptotic response in PDAC [23].

Despite progress in PDAC therapeutics, treatment efficacy remains profoundly limited by the tumor’s desmoplastic stroma and dense microenvironment, which impede drug delivery and sustain intrinsic resistance mechanisms [68]. Standard regimens such as gemcitabine or FOLFIRINOX yield only modest survival improvements, as the tumor microenvironment suppresses apoptosis through pathways like fibronectin-mediated ERK1/2 signaling and connective tissue growth factor-dependent inhibition of caspase activity [69, 70]. These adaptive barriers, encompassing both the physical impediments of the stroma and the molecular survival mechanisms that maintain apoptotic resistance, will likely require not only rational combination strategies but also optimized drug design to achieve durable responses. In our study, the fluorinated Mito-Met_10_ analog demonstrated preferential intratumoral accumulation with minimal liver localization, supporting its potential for enhanced tumor targeting and reduced off-target exposure [22]. Additional safety evidence from renal injury models demonstrates that mitochondria-targeted antioxidants can prevent cisplatin-induced nephrotoxicity without compromising anti-tumor efficacy, underscoring the translational potential of this drug class [71, 72]. These findings suggest that medicinal chemistry refinement of Mito-Met_10_ analogs could yield agents that combine enhanced potency with improved safety profiles relative to other metabolic inhibitors [65].

Taken together, our results reinforce mitochondrial dysfunction-driven ER stress as a tractable vulnerability in PDAC. By exploiting tumor-specific features such as mitochondrial hyperpolarization and dependence on GRP78-mediated proteostasis, Mito-Met_10_ provides a promising therapeutic platform. Future studies will determine whether combining Mito-Met_10_ with other metabolic inhibitors enhances efficacy by fine-tuning UPR activation and apoptotic pathways. Co-targeting mitochondrial bioenergetics and ER stress may produce synergistic anti-tumor effects in PDAC. Additionally, the tumor-extrinsic effects of Mito-Met_10_ and its fluorinated analog *p*CF_3_-Mito-Met_10_ should be explored in vivo, particularly their potential impact on the immune tumor microenvironment and chemotactic signaling. Together, these investigations will clarify both the intrinsic and extrinsic mechanisms by which Mito-Met_10_ exerts its anti-tumor activity.

## Materials and methods

### Cell culture

The human pancreatic carcinoma cell line MiaPaCa-2 (RRID: CVCL_0428) was purchased from the American Type Culture Collection (ATCC, Rockville, MD, USA). The human pancreatic stellate cells HPSC (RRID:CVCL_SA58) were a kind gift from Rosa F. Hwang, MD, MD Anderson Cancer Center, Houston, TX, USA [73]. Murine pancreatic cancer cell lines, KPC1199, KPC 1242, and KPC1245, were derived from C57BL/6 KPC transgenic mice backcrossed to the C57BL/6 genetic background and that spontaneously developed pancreatic tumors and were the kind gift of David Tuveson, MD/PhD (Cold Spring Harbor Laboratory, NY, USA). All cells were cultured in high-glucose DMEM (Gibco Life Technologies, Carlsbad, CA, USA; Cat. #11965084) supplemented with 10% (v/v) fetal bovine serum (FBS; Omega Scientific, Inc., Tarzana, CA, USA; Cat. #FB-21). Immortalized human pancreatic duct epithelial cells hTERT-HPNE (RRID:CVCL_C466) were cultured in DMEM supplemented with 200 mM L-glutamine, 7.5% (w/v) NaHCO_3_, 15% (v/v) FBS, M3-Base A (Incell, San Antonio, TX, USA; Cat.#M300F-500), 1 M D-glucose (Gibco Life Technologies, Carlsbad, CA, USA; Cat.#J60067.EQE), 100 μg/mL human EGF (Gibco Life Technologies, Carlsbad, CA, USA; Cat.#PHG0313), and 10 mg/mL puromycin (Thermo Fisher Scientific, Waltham, MA, USA; Cat.#A1113802). A deidentified patient-derived pancreatic cancer cell line (MCW512) was obtained from the LaBahn Pancreas Cancer Program and the Medical College of Wisconsin Surgical Oncology Biobank using IRB-approved protocols and cultured in RPMI media (Gibco Life Technologies, Carlsbad, CA, USA; Cat.# 11875119) supplemented with 10% (v/v) fetal bovine serum (FBS; Omega Scientific, Inc., Tarzana, CA; Cat.#FB-21). All cell lines were stored in liquid nitrogen and serially passaged at a density of 0.5–1.5 × 10^6^ cells/mL twice per week for a maximum of 20 passages after thawing to ensure experimental consistency. Cells were validated annually using short tandem repeat profiling and mycoplasma-tested semiannually.

### General procedure for the preparation of compounds

The synthesis of Mito-Met_10_ and its fluorinated variant, *p*CF_3_-Mito-Met_10_, were refined from our prior work [22, 74] to increase yield as outlined in Supplementary Fig. 1. The purity was confirmed by ^1^H-NMR and further verified by high-resolution MALDI MS analysis using Bruker timsTOF fleX MALDI-2 system. Both compounds were stored at −80 °C. Samples for MS analysis were prepared at 5 mg/mL in methanol as a stock solution. Equal 1 µL volumes of sample solution [~5 μg] and matrix solution were combined and 1 µL aliquots [~2.5 μg] spotted in duplicate onto the MALDI target plate to assess the purity. DHBA (2,5-dihydroxybenzoic acid) and THAP (2,4,6-trihydroxyacetophenone) were used to evaluate ionization efficiency for MALDI-MS analysis of both compounds.

#### 10-Hydroxydecylbiguanide (1)

10-Amino-1-decanol (2.00 g, 10.60 mmol) was dissolved in pyridine (10.6 mL). This was treated with (E)-*N’*-carbamimidoyl-1H-pyrazole-1-carboximidamide hydrochloride (2.76 g, 15.90 mmol) [75]. The reaction was then heated to 40 °C for 20 h. The reaction was concentrated in vacuo and then azeotroped with toluene (3 ×75 mL) to remove the residual pyridine. The residue was triturated with Et_2_O (5 ×75 mL), and the solids were collected by vacuum filtration and washed with Et_2_O (3 ×25 mL) to give 1 (2.70 g, yield: 99%). MS (ESI) *m/*z 258.2 [M + H]^+^. ^1^H NMR (500 MHz, MeOD) δ 3.54 (t, *J* = 6.6 Hz, 2H), 3.31 (t, *J* = 1.7 Hz, 1H), 3.20 (t, *J* = 7.3 Hz, 1H), 2.63 (t, *J* = 7.3 Hz, 1H), 1.52 (m, 4H), 1.33 (m, 14H). ^13^C NMR (126 MHz, MeOD) δ 42.47, 33.62, 33.54, 30.65, 30.60, 30.55, 30.37, 27.99, 27.86, 26.92.

#### 10-Iododecyl bisguanide (2)

Triphenylphosphine (2.49 g, 9.32 mmol) and imidazole (0.688 g, 10.10 mmol) were dissolved in CH_2_Cl_2_ (40 mL) and placed under nitrogen. The reaction was then cooled to 0 °C in an ice bath and stirred for 5 min. It was then treated with iodine (2.37 g, 9.32 mmol) and stirred at 0 °C for 15 min. The reaction was then treated with 1-hydroxydecyl-bisguanide **1** (2.00 g, 7.77 mmol). The yellow reaction was stirred at 0 °C for 30 min, then at room temperature for 18 h, then concentrated in vacuo. The crude product was purified *via* flash column chromatography on silica gel (CH_2_Cl_2_/MeOH, 98/2 to 80/20) to give **2** (2.30 g, yield: 81%). MS (ESI) *m/*z 368.2 [M + H]^+^.

#### (10-(3-Carbamimidoylguanidino)decyl)tris(4-(trifluoromethyl)phenyl)phosphonium bromide (3) (pCF_3_-Mito-Met_10_)

1-Iododecyl bisguanide **2** (2.09 g, 5.70 mmol) was dissolved in anhydrous CH_3_CN (23 mL) and placed under nitrogen in a pressure bottle. This was treated with tris(4-(trifluoromethyl)phenyl)phosphine (2.66 g, 5.70 mmol). The hazy solution was then heated to 90 °C for 4 h, then at 140 °C for 18 h. The reaction was then concentrated in vacuo. The crude material was dissolved in CH_2_Cl_2_ (50 mL). This was partitioned with a 15% (v/v) aqueous solution of NaBr (2 ×50 mL) and vigorously stirred. The layers were separated, and the CH_2_Cl_2_ layers were combined and dried (Na_2_SO_4_), filtered, and concentrated in vacuo. The crude product was purified *via* flash column chromatography on amine-treated silica gel (CH_2_Cl_2_/MeOH, 99/1 to 70/30) to give **3** (2.82 g, yield: 63%). MS (ESI) *m/*z 706.4 [M - Br]^+^. HRMS (MALDI) m/z: [M]^+^ Calculated for C_33_H_38_N_5_F_9_P 706.2716; Found 706.2715. The spectroscopic data agreed with those already reported [22, 74].

#### 10-Bromodecylbiguanide (4)

10-Hydroxydecylbiguanide **1** (3.94 g, 15.3 mmol) was charged to a pressure vessel and treated with 48% (v/v) aqueous hydrobromic acid (8.0 mL, 70.3 mmol). The reaction was heated to 110 °C for 6 h. The reaction was cooled to room temperature and concentrated in vacuo. The residue was neutralized with sat. NaHCO_3_. This was then extracted with EtOAc (3 ×75 mL). The combined organic layer was washed with brine (1 ×100 mL), then dried (Na_2_SO_4_). It was filtered and concentrated in vacuo to give crude **4**. The crude product was purified *via* flash column chromatography on amine-treated silica gel (CH_2_Cl_2_/MeOH, 94/6 to 50/50) to give **4** (1.79 g, yield: 36.5%). ^1^H NMR (500 MHz, MeOD) δ 3.55 (t, *J* = 6.6 Hz, 1H), 3.44 (t, *J* = 6.7 Hz, 2H), 3.19 (q, *J* = 7.9 Hz, 2H), 2.95 - 2.86 (m, 1H), 2.32 (s, 1H), 1.84 (p, *J* = 6.9 Hz, 2H), 1.75 (p, *J* = 6.9 Hz, 1H), 1.56 (t, *J* = 7.0 Hz, 3H), 1.44 (t, *J* = 7.3 Hz, 3H), 1.34 (d, *J* = 4.3 Hz, 9H). ^13^C NMR (126 MHz, MeOD) δ 45.75, 34.43, 33.98, 33.80, 30.56, 30.51, 30.37, 29.82, 29.15, 27.87.

#### (10-(3-Carbamimidoylguanidino)decyl)triphenylphosphonium bromide (5) (Mito-Met_10_)

10-Bromodecylbiguanide **4** (0.100 g, 0.312 mmol) was dissolved in anhydrous CH_3_CN (1 mL). This was treated with triphenylphosphine (0.082 g, 0.312 mmol), heated at reflux for 19 h, and then concentrated in vacuo. The residue was purified *via* flash column chromatography on amine-treated silica gel (CH_2_Cl_2_/MeOH, 97/3 to 70/30) to give **5** (0.088 g, yield: 48.4%). MS (ESI) *m/*z 502.2 [M - Br]^+^. The ^1^H and ^13^C NMR data agreed with those previously reported [26].

### Animal models

All animal experiments were approved by the Institutional Animal Care and Use Committee (IACUC) at the Medical College of Wisconsin (AUA000076). An orthotopic syngeneic engraftment model was employed to assess tumor progression following treatment with Mito-Met_10_. Male C57BL/6J mice (6-to-8-week-old; RRID: IMSR_JAX:000664) were purchased from The Jackson Laboratory (Bar Harbor, ME, USA). Mice were anesthetized with isoflurane and orthotopically engrafted with 2.5 × 10^5^ KPC 1242 cells into the tail of the pancreas, as previously described [76–78]. Mice were randomly assigned to control or experimental treatment groups immediately prior to treatment in a randomized manner. Five days post-implantation, tumor-bearing mice received a single intraperitoneal injection of compound resuspended in Dulbecco’s-modified PBS (Gibco Life Technologies, Carlsbad, CA, USA ; Cat.#14190) in a total volume of 200 μL. Injections were alternated between flanks using a 1.5 mL syringe and a 28-gauge × 0.5 cm needle (Franklin Lakes, NJ, USA; Cat.#329461). Mice were treated daily for five consecutive days with two days off between cycles. Treatment groups received 3.5 mg/kg *p*CF_3_-Mito-Met_10_, or 2.5 mg/kg or 5 mg/kg unlabeled Mito-Met_10_, while the control group received the vehicle (PBS). Positive control mice were administered 450 μg of DMXAA 5,6-dimethyl-9-oxo-9H-xanthene-4-acetic acid (DMXAA) (Tocris Bioscience, Minneapolis, MN, USA; Cat.#5601) in 50 μL of 0.67% (v/v) NaHCO3-phosphate-buffered saline (PBS) (Thermo Fisher Scientific, Cat#14190-144) on days 12, 15, 19. At the conclusion of the study (day 21), tumors were excised, and their volume measured (length × width × depth = mm³) postmortem using calipers. The maximal tumor diameter permitted by the Institutional Animal Care and Use Committee at the Medical College of Wisconsin is 2 cm. This limit was not exceeded in any of our studies. Investigators performing tumor excisions and measurements were blinded to treatment allocations. Tumors were then fixed in zinc formalin, cleared in 70% (v/v) ethanol, embedded in paraffin for immunohistochemical analyses.

MALDI/TOF imaging was performed on 10 μm fresh frozen tumor sections from mice treated with 3.5 mg/kg *p*CF_3_-Mito-Met_10_ (*n* = 4) or vehicle (*n* = 3), as well as liver tissues from *p*CF_3_-Mito-Met_10_-treated (3.5 mg/kg; *n* = 1) and vehicle-treated (*n* = 1) mice. An additional subcutaneous heterotopic tumor was treated via intratumoral injection of *p*CF_3_-Mito-Met_10_ (3.5 mg/kg; *n* = 1). Tumors were flash-frozen on day 21, two days following the final treatment. All subsequent MALDI-MS analyses were conducted by investigators independent from those performing the in vivo treatments and excisions.

### Migration assay

Cell migration was assessed using 8.0 μm pore size transwell system (6.5 mm diameter, Cat. #3422, Corning, NY, USA). KPC 1242 cells (2 × 10^4^) were serum-starved for 4–6 h prior and seeded into the upper transwell chamber. The lower transwell chambers were filled with 700 μL of serum-free medium or medium containing 10% FBS in the presence or absence of Mito-Met_10_ (8 μM). Cells were allowed to migrate at 37°C with 5% CO_2_ for 8 h. Following incubation, non-migrated cells were removed from the upper membrane surface using a moistened cotton swab. Cells that migrated to the lower chamber were collected by combining bottom well medium, 1X PBS wash, and TrypLE dissociation wash. Suspensions were centrifuged at 300 × *g* for 5 min and resuspended in flow buffer. Viable migrated cells were quantified by flow cytometry (Novocyte Advanteon VBR V8B7R4 (RRID:SCR_019522; Agilent Technologies San Diego, CA, USA) following staining with a fixable viability dye. Migration was expressed either as total viable migrated cells or as a normalized migration index relative to vehicle control.

### Bulk sequencing

RNA samples were harvested from cultured MCW512 cells using the Qiagen RNeasy Mini Kit (Qiagen 74106) following the manufacturer’s instructions. Two biological replicates were prepared per condition: 6 h treatment with vehicle (PBS) or 1 μM Mito-Met_10_. RNA was sent to the Mellowes Center for Genomic Sciences and Precision Medicine at the Medical College of Wisconsin (RRID:SCR_022926) for sample processing and sequencing. mRNA was enriched through poly(A) selection and sequenced using Illumina HiSeq 2500 sequencer to generate paired end reads. The resulting FASTQ files were processed using the nf-core [79] RNAseq 3.12.0 pipeline (10.5281/zenodo.1400710) with default settings and with the hg38 human genome as a reference and Salmon [80] as the pseudo aligner. The raw count matrix was imported into R Studio version 2024.12.1 + 563 and R 4.4.3 and processed by DESeq2 [81] following the vignette for normalization and differential gene expression. The log fold change corrected differentially expressed genes were used to generate the GO terms using the enrichGO package within clusterProfiler [82] with the top 2000 genes. All differentially expressed genes underwent Reactome [83] analysis for further confirmation. The code for the analysis can be found at https://github.com/The-Michael-Dwinell-Lab-at-MCW/Mito-Metformin-Bulk-seq-Analysis/tree/main.

### Transmission electron microscopy

MiaPaCa-2 cells (1 × 10^6^) were treated with vehicle (DMSO, Sigma-Aldrich, Saint Louis, MO, USA; Cat.#D2650-100ML), Mito-Met_10_ (0.3 μΜ), or FCCP (Sigma-Aldrich; Cat.#C2920-10MG) (20 μΜ) while 1 × 10^6^ HPNE cells were treated with vehicle (DMSO), Mito-Met_10_ (10 μΜ), or FCCP (20 μΜ) for 6 h. Cells were washed, fixed in situ with 4% (w/v) paraformaldehyde +2% (v/v) glutaraldehyde in 0.1 M sodium cacodylate buffer for 10 min at 22 °C. Cells were gently scraped from the culture dishes, pelleted, washed with 3 × 10 min rinses in 0.1 M cacodylate buffer and postfixed in potassium ferricyanide reduced 1% (v/v) osmium tetroxide for 2 h on ice. Cell pellets were processed into epoxy resin (EMBed 812). 60 nm sections were stained with uranyl acetate and lead citrate and imaged on a Hitachi H600 TEM operated at 75 kV. Images were acquired using an ORCA camera (448 ms exposure, gain 1, binning 1) under standard contrast settings. Representative images were collected at direct magnifications ranging from ×5000 to ×20,000, corresponding to scale bars of 2 μm, 1 μm, and 500 nm. Multiple non-overlapping fields were imaged per condition. Images were randomized and graded for mitochondrial damage by an investigator blinded to the experiment. Grading was completed by identifying the total number of mitochondria and how many exhibited vacuolization, concentric onion-shaped cristae, branching, and mitophagy as previously described [35]. Enumerated damaged mitochondria divided by the number of total mitochondria to get a percent of damage and a percent of healthy mitochondria. Score of minimal (0) = 0–24.99%, mild (1) = 25–49.99%, moderate (2) = 50–74.99%, severe = 75–100% (3). Mitochondria that were out of focus were excluded from the measurements as damage could not be verified morphologically.

### Confocal imaging

MiaPaCa-2 cells (2.5 × 10^5^) were seeded onto 24-well glass-bottom plates (Cellvis P24-1.5H-N) pre-coated with poly-D-lysine to enhance attachment. Cells were treated with vehicle or Mito-Met_10_ (0.3 μΜ) for 6 h. For mitochondrial depolarization controls, cells were treated with FCCP (20 μΜ) for 30 min prior to staining. Cells were incubated with MitoTracker Red CMXRos (300 nM; Invitrogen, CA, USA, Cat#: M7512) for 30 min at 37 °C, followed by nuclear staining with Hoechst as previously described [84]. After washing with pre-warmed PBS, live cells were imaged using an Andor BC43 confocal microscope (Oxford Instruments, Abingdon, UK) with a 100X objective. Images were acquired under identical exposure and laser settings across conditions and processed uniformly. Fluorescence intensity was quantified from whole field measurements using FIJI open-source imaging software (RRID:SCR_002285). Two to three fields were analyzed and averaged for each biological replicate.

### TMRE

MiaPaCa-2, KPC 1242, and HPNE (5 × 10^5^) were treated with FCCP (20 μΜ) for 15 min or increasing concentrations of Mito-Met_10_ for 6 h and 24 h. TMRE (200 nM) was added for 30 min before harvesting for analysis on a Cytek Aurora flow cytometer (Cytek Biosciences, Fremont, CA, USA; RRID:SCR_019826) and data analyzed using FlowJo software (FlowJo, LLC, Ashland, OR,USA).

### Seahorse extracellular flux analysis

Mitochondrial respiration was measured using a Seahorse XF96 Extracellular Flux Analyzer (Agilent Technologies, Santa Clara, CA, USA). Cells were seeded in Seahorse XF96 cell culture microplates at 25,000 cells per well in 200 μL RPMI medium and incubated at 37 °C with 5% CO_2_ for 24 h. Cells were pre-treated with vehicle or Mito-Met_10_ for 4 h prior to assay. On the day of the assay, growth medium was replaced with serum-free assay medium and plates were equilibrated in a non-CO_2_ incubator at 37 °C for 45–60 min before measurement. Oxygen consumption rate (OCR) and Extracellular acidification rate (ECAR) were measured under basal conditions and following sequential injection of oligomycin (Oligo, 1.5 μM), FCCP (1 μM), and rotenone/antimycin A (Rot/AA, 0.5 μM). Basal respiration and ATP-linked respiration were calculated as previously described [85].

### Immunoblotting

Human and mouse PDAC cell lines (1.2 × 10^6^) were plated to 6-well plates and stimulated with titrated doses of Mito-Met_10_ for 30 min, 2.5 h, 8 h, 12 h, 16 h, 24 h, or 48 h in full growth medium. After stimulation, cells were washed, lysed using a modified radioimmunoprecipitation assay (RIPA) buffer containing protease inhibitors (aprotinin, leupeptin, phenylmethylsulfonyl-fluoride and pepstatin) and phosphatase inhibitor cocktail on ice for 10 min. Lysates were clarified by centrifugation at 8000 x *g* for 10 min at 4 °C. Total protein concentration of lysates was determined by the Lowry method [86]. Cellular lysates (25 μg) combined with an equal volume of sample treatment buffer containing 5% (v/v) 2-mercaptoethanol were boiled for 5 min at 95 °C, separated by 10% (v/v) SDS-PAGE, and wet-transferred to a nitrocellulose membrane. The membranes were blocked in 5% milk or BSA in tris-buffered saline with tween (TBST) at room temperature and then incubated with the primary antibody in 1% (w/v) milk or 1% (w/v) BSA overnight at 4 °C. Antibodies against BiP (C50B12) (Cat.#3177), ATF4 (D4B8) (Cat.#11815), phospho-eIF2α (Ser51) (Cat.#9721), and eIF2α (Cat.#9722) were purchased from Cell Signaling Technology (Danvers, Massachusetts, USA). Antibody to ATF6 (3B7E4) (Cat. #66563-1-IG) was purchased from ThermoFisher Scientific. Membranes were washed 3 times in TBST at room temperature and then incubated with horseradish peroxidase-conjugated secondary antibody in 1% (w/v) milk at room temperature for 1 h. SuperSignal West Pico (Thermo Fisher Scientific Cat.#34580) or Fempto Chemiluminescent Substrate (Thermo Fisher Scientific, Cat.#34094) was allowed to react with membranes for 5 min before blots were imaged using a ChemiDoc MP (BioRad, Hercules, CA, USA; Cat.#12003154). The intensity/optical density values of the bands were measured using Bio-Rad Image Lab Software (Version 6.1.0 build 7) and normalized to a housekeeping protein (β-actin).

### Quantitative real-time RT-PCR (qPCR)

Human and mouse PDAC cell lines (1 × 10^6^) were plated to 6-well plates and stimulated with Mito-Met_10_ or respective inhibitors for indicated time points in full growth medium. Total RNA was extracted using the RNeasy™ Mini Kit (QIAGEN, Aarhus, Denmark; Cat. #74106) following the manufacturer’s instructions. Total RNA was quantified spectrophotometrically using a NanoDrop™ 2000 Spectrophotometer (Thermo Scientific, Cat. #ND-2000). From total RNA, 2 µg per sample was reverse-transcribed into single-stranded cDNA using the High-capacity cDNA reverse transcriptase kit (Thermo Fisher Scientific, Cat#4368814) following manufacturer’s instructions on a Mastercycler® nexus X2-PCR Thermal Cycler (Eppendorf, Framingham, MA, USA; Cat. # E6338000020). The resulting cDNA was subjected to real-time RT-PCR quantification with SsoAdvanced Universal SYBR Green Supermix (BioRad, Cat. #1725271) on CFX Opus 96 Real-Time PCR System (Bio-Rad). Thermal conditions for a final reaction volume of 10 μl with an initial denaturation at 95 °C for 3 min followed by 40 cycles of 95 °C for 10 s, annealing and extension at 60 °C for 30 s in 96-well reaction plates (Bio-Rad; Cat. #64647348). The polymerase chain reaction (PCR) was performed in technical triplicate for each sample, with a negative control well with no template control. All experiments were repeated in at least three independent biological replicates and collected on CFX Maestro 2.3 software (BioRad; version 5.3.022.1030). Cycle threshold (C_t_) values were normalized for amplification using β-actin, and data analyzed using the 2^-ΔΔCt^ method on GraphPad Prism10.4.2 (GraphPad Software, San Diego, CA; RRID: SCR_002798). Primers were designed and purchased from IDT Technologies (Coralville, IA, USA) and sequences are detailed in Supplementary Table 1.

### Live cell growth and apoptosis

Cell line sensitivity to Mito-Met_10_ was evaluated in multiple rounds of screening, with cell lines showing differential responses advancing to further testing. To generate concentration-response curves, cells were seeded to individual 96-well plates (Techno Plastic Products, Switzerland, Cat. # 92696) at densities that would allow them to grow to 80–90% confluence in 72 h in 100 μl of growth medium per well. Twenty-four hours after plating, cells were treated with 100 μl of full growth medium containing twice the final desired concentration of Mito-Met_10_. The range of concentrations used for each cell line can be found in Supplementary Table 2. Concentrations were selected to span a broad dynamic range, from sub-micromolar to higher micromolar levels, to capture both early stress responses and overt cytotoxic effects. This design also enabled comparison of sensitivity between malignant and non-malignant pancreatic cells. Each dose response curve had four technical replicates per cell line. Cell confluence was measured using IncuCyte S3 Live Cell Analysis System (Sartorius, Ann Arbor, MI, USA) scanned every 4 h with the 10x microscope objective.

To measure apoptosis, cells were plated at a density of 4 × 10^4^/well in 100 μl, with a minimum of 4 technical replicates per experiment. After 24 h cells were treated with respective concentrations of Mito-Met_10_. Incucyte caspase-3/7 green dye (1:1000) (Sartorius, Ann Arbor, MI, USA; Cat. #4440) was added to individual wells and cells imaged using the IncuCyte S3 imaging software system (Sartorius, Ann Arbor, MI, USA) using a 10x objective every 4 h. The caspase-3/7 reagent used in these experiments is cell membrane permeable and non-toxic, allowing live-cell visualization of apoptotic progression. This reagent contains a DNA-binding dye linked to a DEVD peptide substrate that prevents DNA-binding until cleaved by active caspase-3/7, upon which the dye translocates to the nucleus, binds DNA, and emits fluorescence. The Incucyte analysis system was used to quantify caspase-3/7 activity normalized to cell confluence at each time point and concentration. Proliferation rates were determined as previously described [34].

For metabolite rescue experiments, the same protocol was followed with the addition of exogenous metabolites. Twenty-four hours after seeding, cells were washed with sterile PBS and cultured in growth medium supplemented with L-aspartic acid (10 mM; Sigma Aldrich, Saint Louis, MO, USA Cat#: A9256) or sodium pyruvate (2 mM, Agilent, CA, USA, Cat#:103578). Supplemented media were sterile filtered through a 0.22 μm filter prior to use.

In selected experiments, cells were treated with the following pharmacologic inhibitors: z-Val-Ala-Asp-fluoromethylketone (z-VAD-fmk; 5 μM; InvivoGen, San Diego, CA, USA; Cat#:tlrl-vad), a pan-caspase inhibitor; necrostatin-1 (Nec-1; 20 μM; Enzo Life Science Inc., Farmingdale, NY, USA; Cat#:BML-AP309-0020), a RIP1 inhibitor; integrated stress response inhibitor (ISRIB; 500 nM; Sigma-Aldrich, Saint Louis, MO, USA; Cat#: SML0843); and thapsigargin (1.5 μM; MedChemExpress, NJ, USA; Cat#:hy-13433). All compounds were prepared according to manufacturer instructions and diluted to final working concentration in complete growth medium immediately prior to use.

### Apoptosis detection (flow cytometry)

Annexin V-FITC (BioLegend, San Diego, California, USA; Cat. #640906) labeling was performed as indicated by BioLegend. Briefly, MiaPaCa-2, KPC 1242, MCW512 and HPSC cells were plated at a density of 1 × 10^6^ to 6-well plates (Corning, Corning, NY, USA; Cat. #3506), and treated for indicated time points with Mito-Met_10_ or respective inhibitors. Cells were collected from plates using warmed TrypLE Express (Thermo Fisher Scientific, Cat #12605028) (5 min at 37 °C followed by TrypLE inhibitor treatment). Cells were then rinsed with warm phosphate-buffered saline and incubated protected from light at room temperature for 15 min in 100 μl of binding buffer (10 mM HEPES, pH 7.4; 140 mM NaCl; 5 mM CaCl_2_) containing 2.5 μl of annexin V-FITC. Labeled cells were washed in binding buffer and DNA content was determined on fixed cells stained with 1 μl (100 μg/ml) of propidium iodide (PI) (Thermo Fisher Scientific, Cat. #P3566) in 100 μl of binding buffer. Samples were analyzed within 1 h of staining on Novocyte Advanteon VBR V8B7R4 (RRID:SCR_019522; Agilent Technologies San Diego, CA, USA) and data were analyzed on NovoExpress flow cytometry software (version 1.6.3). In addition to in vitro Annexin V and PI staining, tumors harvested and excised and dissociated using a Mouse Tumor Dissociation Kit (Miltenyi Biotec, Auburn, CA, USA; Cat. #130-096-730). Dissociated tumors were then stained with Annexin V-FITC and PI.

### MALDI-MS imaging

#### Sample preparation

Place gross tissue into DPBS (Gibco#14190-144) until ready to embed and freeze. Specimens were embedded in 2% (w/v) sodium carboxymethyl cellulose (CMC) (Sigma Cat.# 419273). Tissues were frozen in isopentane chilled in liquid nitrogen and stored at −80 °C. The 10 µm sections were thaw-mounted onto indium tin oxide (ITO)-coated glass slides (Bruker, Billerica, Massachusetts, USA) for imaging mass spectrometry analysis. The mounted tissue slides were stored at −80 °C until analysis. Before matrix application, slides were placed in a desiccator and dried under vacuum for at least 20 min.

#### Matrix application

To evaluate ionization efficiency before tissue imaging, Mito-Met_10_ with and without trifluoromethyl groups (CF_3_) was applied to a stainless steel MALDI plate and analyzed using two matrices, DHBA (2,5-dihydroxybenzoic acid) and THAP (2,4,6-trihydroxyacetophenone). DHBA was chosen for tissue imaging because it demonstrated better ionization efficiency compared to THAP. DHBA (20 mg/mL) was dissolved in 70% (v/v) methanol and applied to the tissue sections using the HTX M3+ Sprayer (HTX Technologies) with the following parameters: temperature: 75 °C, number of passes: 8, flow rate: 100 μL/min, velocity: 1200 mm/min, track spacing: 3 mm, pressure: 10 psi.

#### MALDI-MS imaging

All analyses were performed using a Bruker timsTOF fleX MALDI-2 (RRID: SCR_023615) equipped with a 10 kHz laser. Tissue imaging for MS analysis was conducted in positive ion mode with a mass range of m/z 300–1300. Molecular distribution imaging was set to 20 μm/pixel, with 200 laser shots per pixel at a frequency of 10 kHz.

#### Data processing and imaging

The raw data containing the individual spectra for each measurement region were imported into SCiLS Lab (RRID:SCR_014426) version2024b Pro (Bruker) for molecular imaging. Root mean square normalization was applied for data normalization.

### Immunohistochemistry

Formalin-fixed, paraffin-embedded tumor specimens were processed and sectioned by the Children’s Research Institute Histology Core. Tissue sections were cut and mounted onto Bond 380 slides for automated staining. Immunohistochemical staining was performed using an anti-ATF4 polyclonal antibody (Proteintech, Rosemont, IL, US; Cat#:10835-1-AP) at a dilution of 1:200. Antigen retrieval and staining were performed on a Leica Bond-III automated staining platform according to core facility protocols. Endogenous peroxidase activity was quenched with hydrogen peroxide for 10 min. Detection was carried out using the Leica Bond Polymer Refine Detection system (LSA aRBT, 1:500). Immunoreactivity was visualized using a chromogenic detection system and counterstained with hematoxylin. Images were acquired at 40X magnification using a Nikon upright microscope. ATF4 staining intensity was evaluated using a semi-quantitative scoring system (0 = negative, 1 = very weak, 2 = moderate, 3 = strong). Scoring was performed in a blinded manner by an investigator independent of treatment group allocation. Statistical comparisons between vehicle-treated and Mito-Met_10_-treated tumors were performed using the Mann–Whitney test.

### Illustrations

All schematics and graphical illustrations were created using BioRender (BioRender.com).

### Statistical analysis

No formal power calculations were performed. Sample sizes for in vivo studies were determined based on feasibility, prior published work using this model, and previous experience with treatment effect magnitude in this model. In vitro experiments were performed with a minimum of three independent biological replicates with at least 2 technical replicates, consistent with standard practice. Samples within a biological replicate were processed in parallel and assigned to treatment conditions without formal randomization. All exclusion criteria were established before data analysis. All statistical analyses were performed using GraphPad Prism version 10.6.1 (GraphPad Software, La Jolla, CA). Prior to statistical testing, datasets were assessed for normality and equality of variances. Statistical tests used for each experiment are specified in the corresponding figure legends. All statistical tests were two-sided. A *p*-value ≤0.05 was set as the threshold for statistical significance. Results are presented as the mean ± standard deviation (SD) or as indicated in the figure legend.

## Supplementary information

Supplemental Information for Publication

Original Data

## Data Availability

All data relevant to the study are included in the article or uploaded as online Supplementary information. The raw and processed files for the bulk RNA sequencing project are available at GSE304335. Any further information about resources and reagents should be directly requested from the corresponding authors and will be fulfilled on reasonable request.
