# Supplementary material for: Mitochondria-targeted metformin analogs activate the ER stress-unfolded protein response pathway to drive apoptosis in pancreatic cancer
Source: Cell Death Dis. 2026 May 22;17(1):643. doi: 10.1038/s41419-026-08859-y (PMC13372815; doi:10.1038/s41419-026-08859-y)
Supplement: Supplementary file 1 — Supplemental Information for Publication [file 41419_2026_8859_MOESM1_ESM.docx]

**SUPPLEMENTARY INFORMATION**

**Mitochondria-targeted metformin analogs activate the ER stress-unfolded protein response pathway to drive apoptosis in pancreatic cancer**

Maria Poimenidou^1,2^, Jordan M. Bobek^3^, Donovan Drouillard^1,2^, Tyler Harris^1^, Elisabeth Solis^1,2^, Chad Darnell^2^, Donna McAllister^1,2^, Robert F. Keyes^3,4^, Mayumi Ishihara-Aoki^5^, Kazuhiro Aoki^5,6^, Daisy Sahoo^2,7^, Balaraman Kalyanaraman^5,8^, Brian C. Smith^3,4,5^, and Michael B. Dwinell^1,2,5,9#^

^1^ Department of Microbiology & Immunology, Medical College of Wisconsin, Milwaukee, WI, 53226 USA

^2^ Center for Immunology, Medical College of Wisconsin, Milwaukee, WI, 53226 USA

^3^ Department of Biochemistry, Medical College of Wisconsin, Milwaukee, WI, 53226 USA

^4^ Program in Chemical Biology, Medical College of Wisconsin, Milwaukee, WI, 53226 USA

^5^ Cancer Center, Medical College of Wisconsin, Milwaukee, WI, 53226 USA

^6^ Department of Cell Biology, Neurobiology, and Anatomy, Medical College of Wisconsin, Milwaukee, WI, 53226 USA

^7^ Department of Medicine, Medical College of Wisconsin, Milwaukee, WI, 53226 USA

^8^ Department of Biophysics, Medical College of Wisconsin, Milwaukee, WI, 53226 USA

^9^ Department of Surgery, Medical College of Wisconsin, Milwaukee, WI, 53226 USA

**Supplementary Figure 1. Synthesis of *p*CF_3_-Mito-Met_10_.**

**
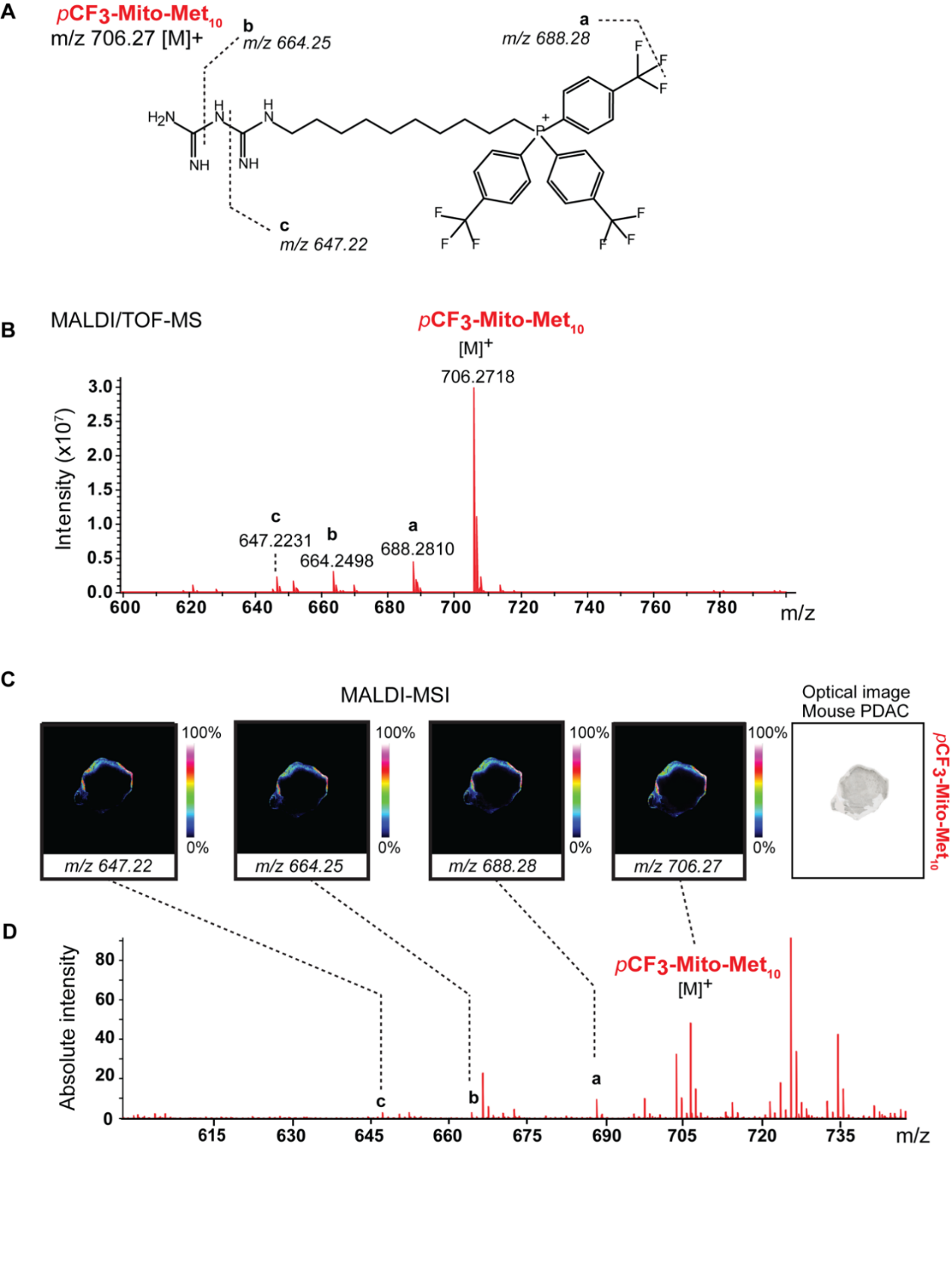
Supplementary Figure 2. Mass spectrometric and imaging characterization of *p*CF_3_-Mito-Met_10_ in murine pancreas tumors.** **(A)** Chemical structure of *p*CF_3_-Mito-Met_10_, precursor mass, and schematic representation of in-source fragmentation sites. The structure indicates the phosphonium cation [M]^+^ of the precursor ion at m/z 706.27, with labeled minor components corresponding to specific regions of the molecule. **(B)** Before tissue imaging, *p*CF_3_-Mito-Met_10_ was spotted onto a conventional stainless steel MALDI plate and analyzed by MALDI/TOF-MS in positive ion mode using a DHBA matrix. The compound ionized efficiently under these conditions, yielding the phosphonium cation [M]⁺ at m/z 706.27 as the primary signal, alongside in-source fragments labeled as *a* (m/z 688.28), *b* (m/z 664.25), and *c* (m/z 647.22). **(C)** An *ex vivo* PDAC mouse tumor, treated intratumorally with *p*CF_3_-Mito-Met_10_, was cryosectioned and subjected to spatial omics analysis using timsTOF fleX MALDI MS imaging. In addition to the phosphonium cation at m/z 706.27, in-source fragments (*a*, *b*, *c*) were detected with the same spatial distribution, indicating their origin from *p*CF_3_-Mito-Met_10_. MALDI-MS images for each ion are shown. **(D)** Mean MALDI-MS imaging spectrum from the *ex vivo p*CF_3_-Mito-Met_10_-treated tumor tissue, highlighting the precursor ion and its in-source fragments.

**
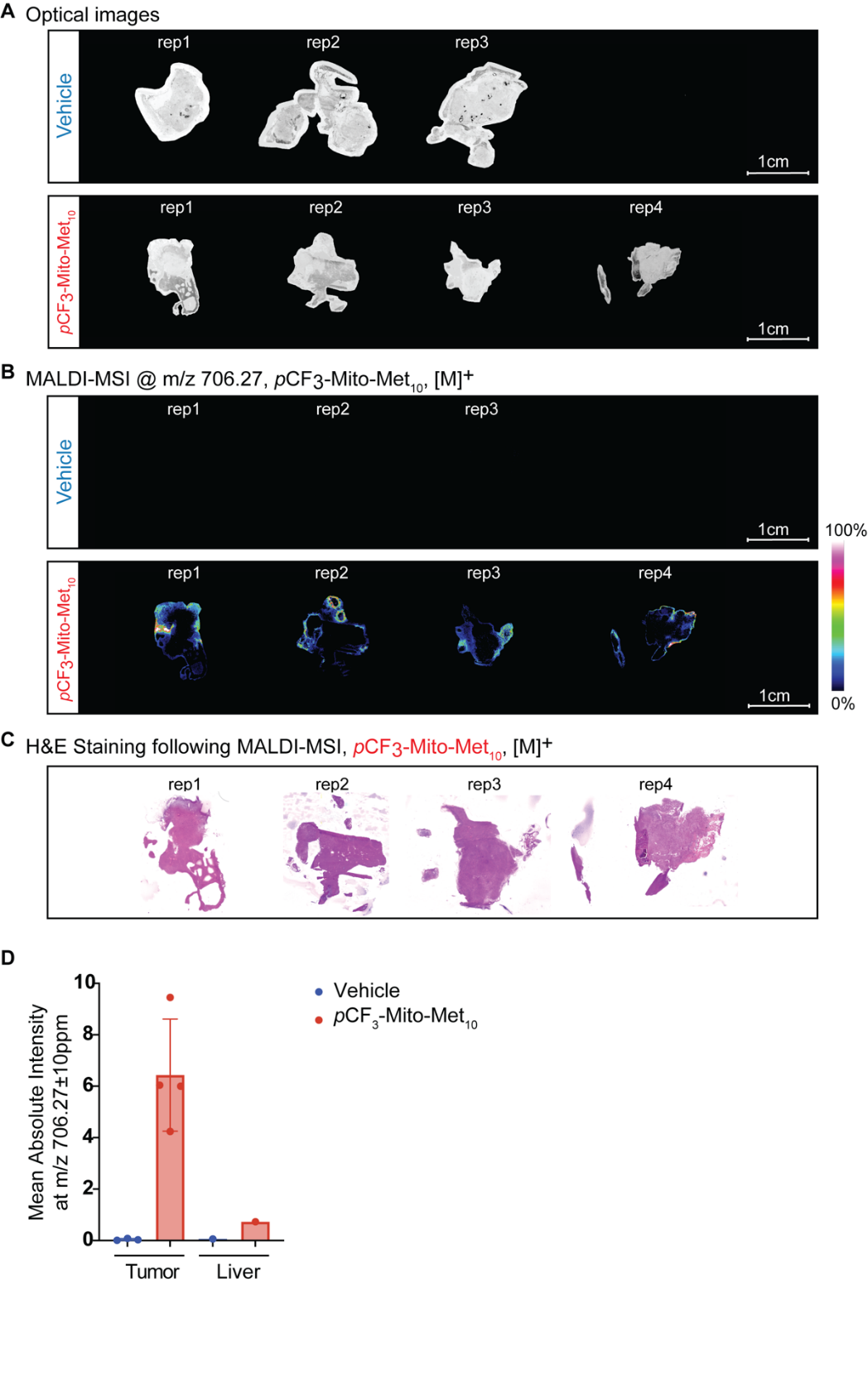
Supplementary Figure 3**. **Visualization and quantitative analysis of *p*CF_3_-Mito-Met_10_** **distribution in PDAC tumors by MALDI MS imaging.** **(A)** PDAC engrafted mice were treated intraperitoneally with either vehicle (n=3) or 3.5 mg/kg *p*CF_3_-Mito-Met_10_ (n=4) for 10 consecutive days. On day 16, tumors were harvested, embedded in 2% CMC, cryosectioned at 10 μm, and mounted on ITO-coated slides. Sections were coated with DHBA matrix and analyzed using timsTOF fleX MALDI-2 in positive ion mode (m/z 300–1300, 20 μm spatial resolution). Optical images of representative tumor sections for each replicate are shown. **(B)** MALDI-MS ion images at m/z 706.27, corresponding to the phosphonium cation [M]⁺ of *p*CF_3_-Mito-Met_10_, from the same tissue sections in panel A. *Top row*: vehicle-treated mice; *bottom row*: pCF_3_-Mito-Met_10_-treated mice. **(C)** Following MALDI MS imaging, tumor sections from treated mice were H&E-stained to confirm tissue morphology and integrity. (**D**) Quantification of *p*CF_3_-Mito-Met_10_ signal intensity in pancreas and liver tissues. Mean absolute intensity of the ion at m/z 706.27±10 ppm in pancreas tumor tissues from vehicle-treated (n = 3) and *p*CF_3_-Mito-Met_10_-treated (n = 4) mice, compared with liver tissue from a single vehicle-treated or *p*CF_3_-Mito-Met_10_–treated mouse. Data are shown as mean ± SD.

**
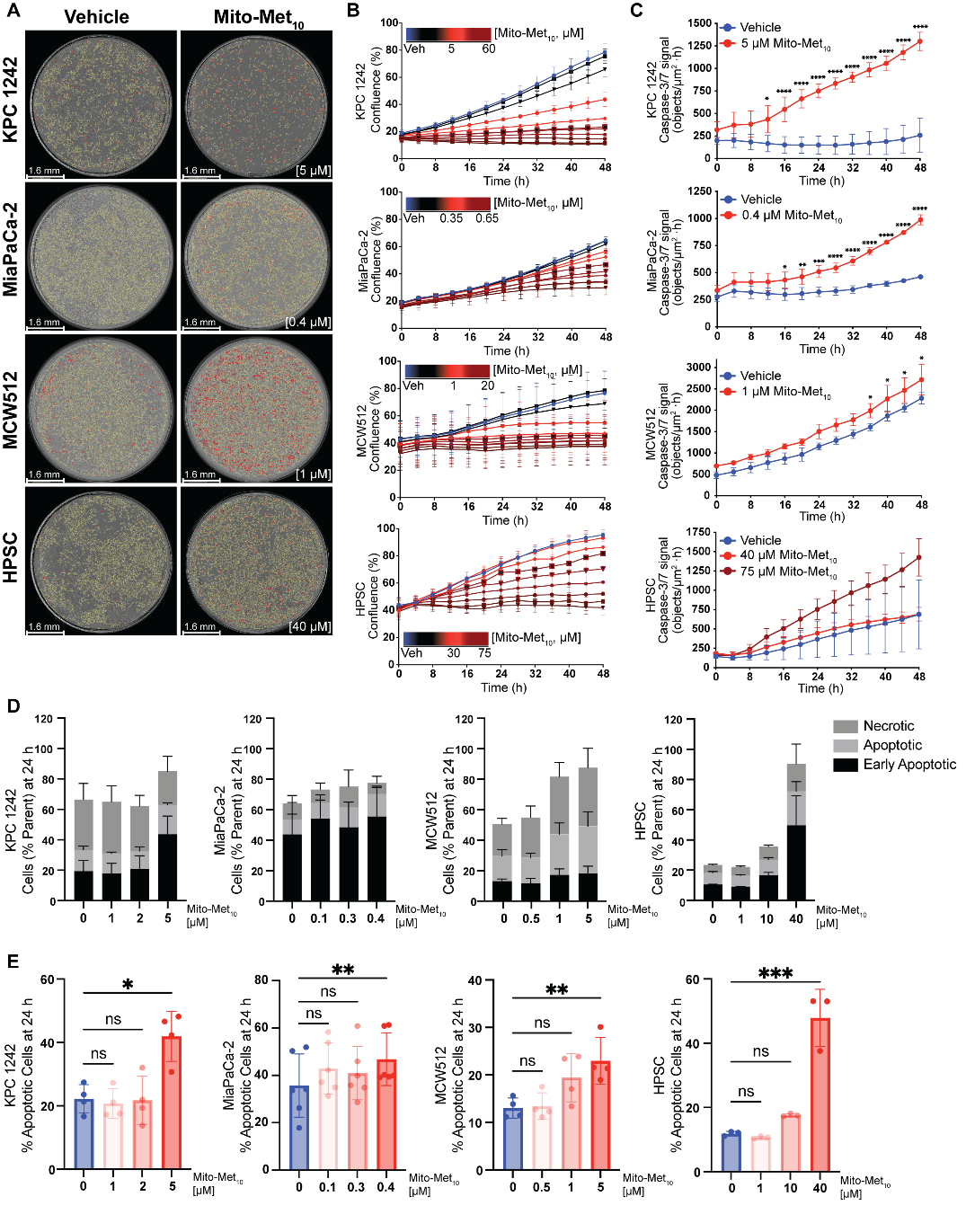
Supplementary Figure 4. Mito-Met_10_-induced apoptosis and growth suppression in pancreatic cell lines. (A)** Representative whole-well images corresponding to those used for Figure 2C, showing vehicle (left) and Mito-Met_10_-treated (right) KPC 1242, MiaPaCa-2, MCW512, and HPSC cells at 48 h at the indicated doses. *Yellow* outlines indicate cell confluence masks generated by IncuCyte analysis, and red fluorescence denotes caspase-3/7-positive apoptotic cells. **(B)** Kinetic proliferation curves showing percent confluence following treatment with the indicated concentrations of Mito-Met_10_ for KPC 1242, MiaPaCa-2, MCW512, and HPSC cells. These data were used to generate IC_50_ curves shown in the main figure. **(C)** Caspase-3/7 activity measured longitudinally by live-cell imaging and quantified as total green object integrated intensity (μm^2^/image) following Mito-Met_10_ treatment in KPC 1242, MiaPaCa-2, MCW512, and HPSC cells. Vehicle and treatment conditions were compared using two-way ANOVA with Sidak’s multiple comparisons test. **(D)** Quantification of necrotic, apoptotic, and early apoptotic cell populations 24 h after vehicle or Mito-Met_10_ treatment in KPC 1242, MiaPaCa-2, MCW512, and HPSC cells. **(E)** Quantification of Annexin V-positive apoptotic cells 24 h after vehicle or Mito-Met_10_ treatment (corresponding to Figure 2E). Apoptotic cells were defined as Annexin V-positive populations following Annexin V-FITC/Propidium iodide staining and flow cytometry analysis. Statistical comparisons were performed within each cell line using repeated-measures one-way ANOVA with Tukey’s multiple comparisons test. Values are mean ± SD. **p*<0.05, ***p*<0.01, ****p*<0.001, *****p*<0.0001, ns = not significant.

**
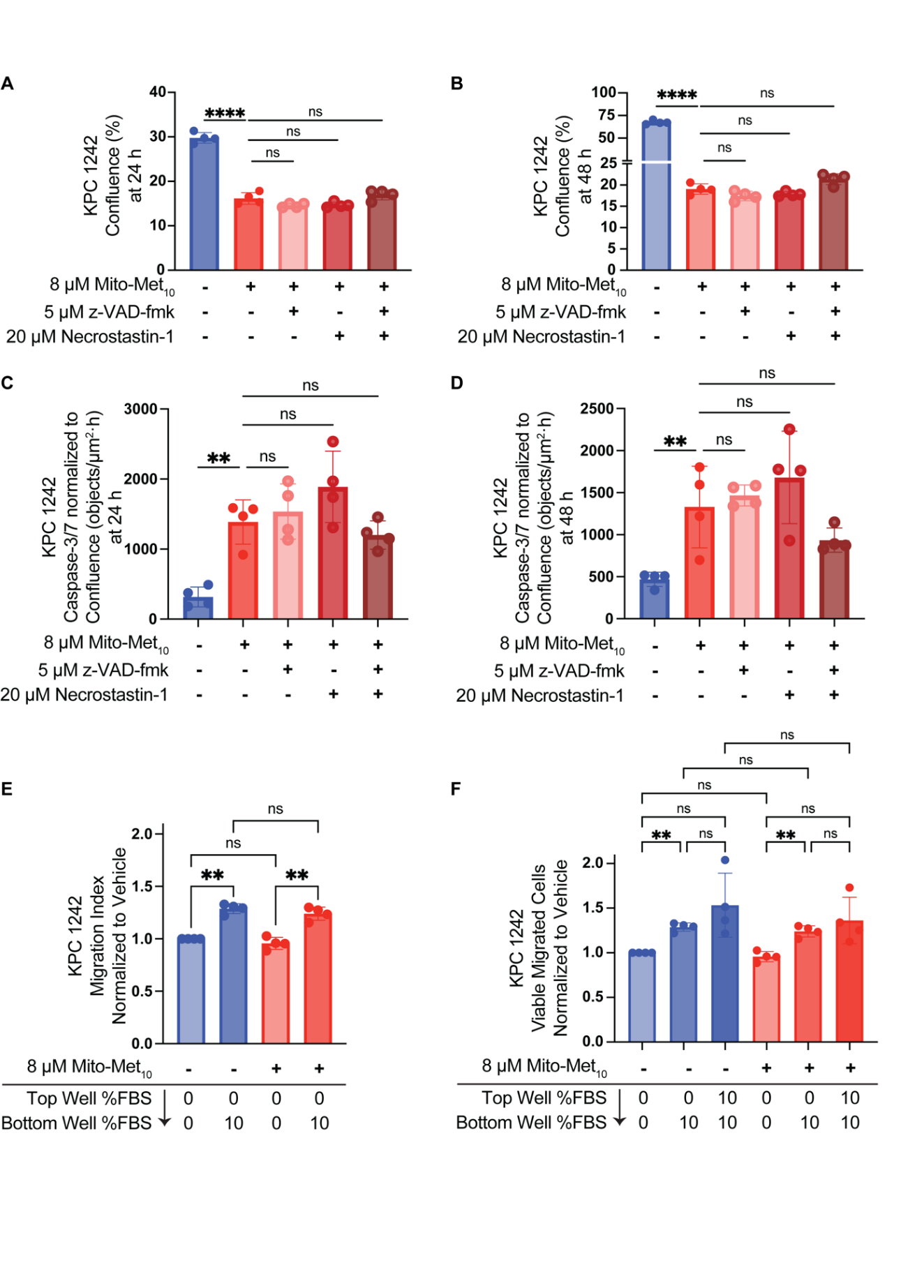
**

**Supplementary Figure 5. Apoptosis inhibition and migration following Mito-Met_10_ treatment in KPC 1242 cells.** **(A-B)** Cell confluence of KPC 1242 measured by live-cell imaging at **(A)** 24 h and **(B)** 48 h following treatment with Mito-Met_10_ (8 μM) alone or in combination with z-VAD-fmk (5 μM), Necrostatin-1 (20 μM), or both inhibitors. Statistical comparisons were performed using ordinary one-way ANOVA with Dunnett’s multiple comparison test. **(C-D)** Caspase-3/7 normalized to confluence at **(C)** 24 h and **(D**) 48 h following treatment with Mito-Met_10_ (8 μM) alone or in combination with z-VAD-fmk (5 μM), Necrostatin-1 (20 μM), or both inhibitors in KPC 1242 cells. Statistical comparisons were performed using ordinary one-way ANOVA with Dunnett’s multiple comparison test. **(E-F)** Transwell chemotaxis assay for KPC 1242 in the presence or absence of Mito-Met_10_ (8 μM) for 8 h. Cells were seeded in serum-free medium (0% FBS) or medium containing 10% (v/v) FBS in the upper chamber, with lower chambers containing either 0% or 10% (v/v) FBS. Migration was quantified as **(E)** normalized migration index relative to vehicle and **(F)** total viable migrated cells. Statistics were analyzed using repeated-measures one-way ANOVA with Tukey’s multiple comparisons test. Values are mean ± SD. **p*<0.05, ***p*<0.01, ****p*<0.001, *****p*<0.0001, ns = not significant.

**
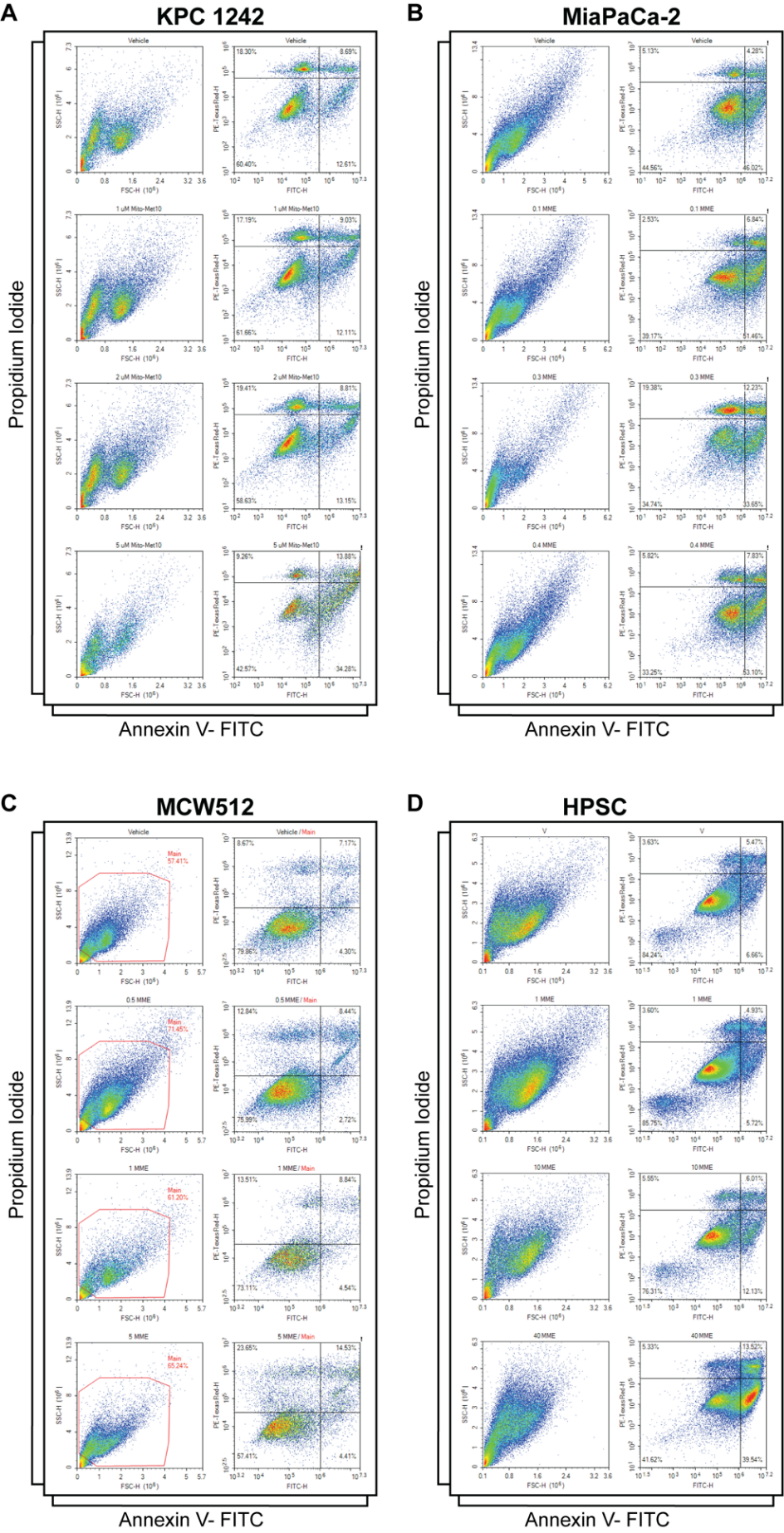
**

**Supplementary Figure 6. Gating scheme for Annexin V-FITC and Propidium Iodide staining.** Representative Annexin V-FITC and PI flow cytometry plots showing gating used to quantify apoptotic populations in **(A)** KPC 1242, **(B)** MiaPaCa-2, **(C)** MCW512, and **(D)** HPSC cells following 24 h treatment with vehicle or increasing concentrations of Mito-Met_10_ (MMe). Gating strategy was used to define viable (Annexin V^-^/PI^-^), early apoptotic (Annexin V^+^/PI^-^), late apoptotic (Annexin V^+^/PI^+^), and necrotic (Annexin V^-^/PI^+^) populations. These representative plots correspond to the quantitative analysis shown in Supplementary Figure 4D-E and Figure 2D-E. Quadrant gates were defined using vehicle controls and applied uniformly across all conditions within each cell line.

**
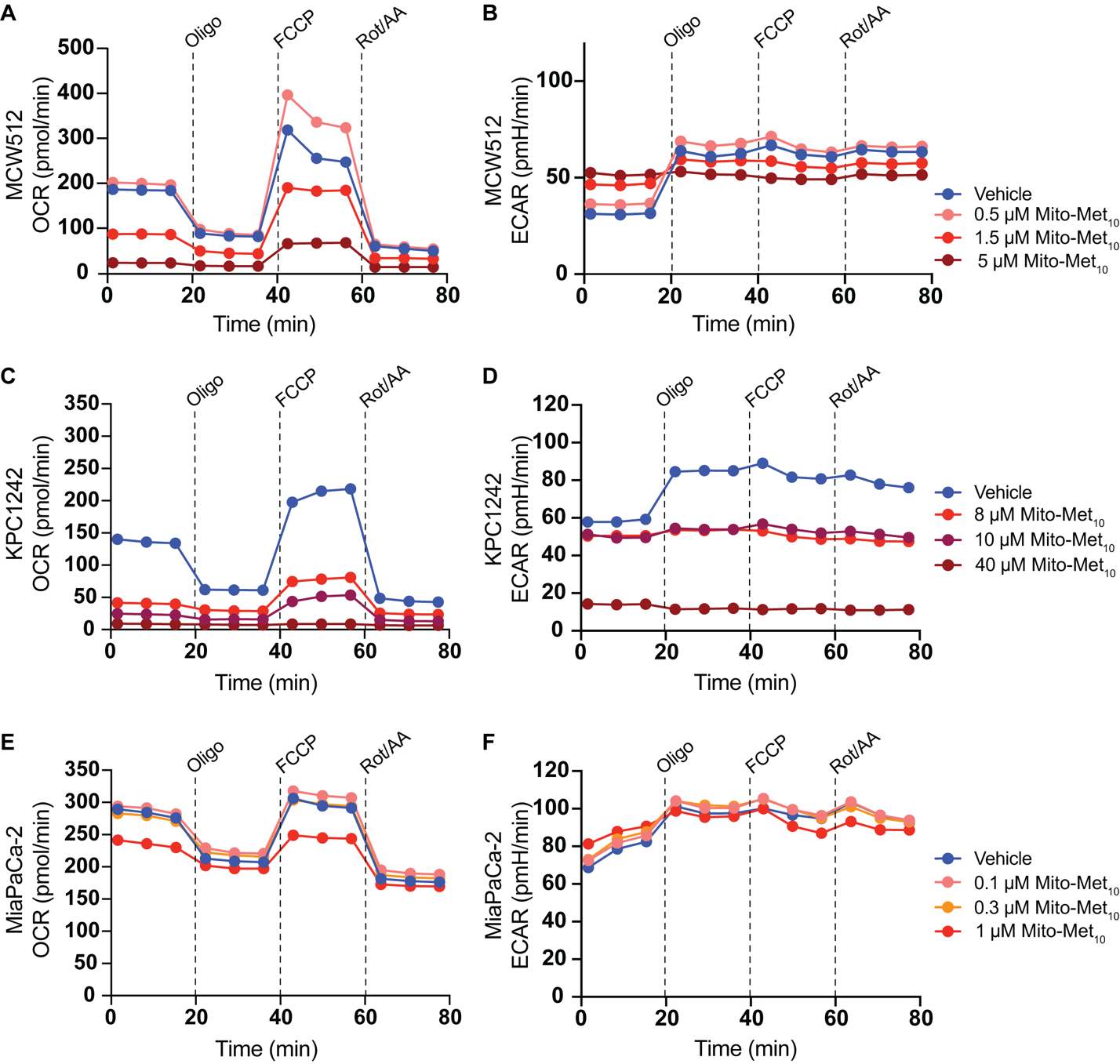
**

**Supplementary Figure 7. Mito-Met_10_ disrupts mitochondrial respiration as an early response to treatment.** Seahorse analysis of oxygen consumption rate (OCR) trace in **(A)** MCW512, **(C)** KPC 1242, and **(E)** MiaPaCa-2 cells following 4 h pre-treatment with increasing doses of Mito-Met_10_. Mitochondrial function was assessed by sequential injection of oligomycin (Oligo, 1.5 μM), FCCP (1 μM), and rotenone/antimycin A (Rot/AA, 0.5 μM). Dashed vertical lines indicate injection times. **(B, D, F)** Extracellular acidification rate (ECAR) trace in respective cell lines.

**
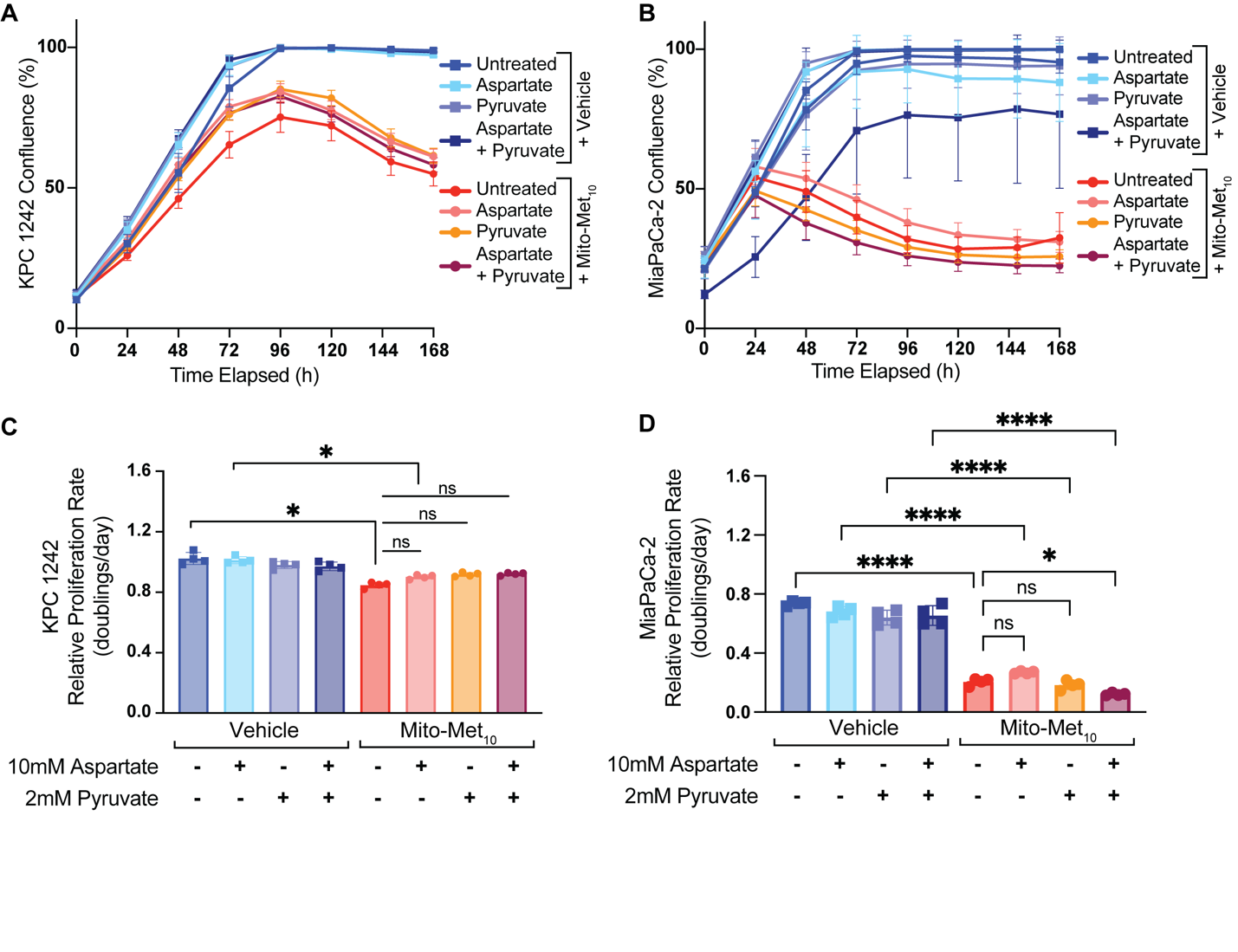
**

**Supplementary Figure 8. Mito-Met_10_-induced growth suppression was not rescued by metabolic supplementation.** PDAC cell confluence of **(A)** KPC 1242 and **(B)** MiaPaCa-2 cells measured over time by live-cell imaging under vehicle (blue curves) or Mito-Met_10_ (red curves) treatment. Cells were cultured in standard medium or supplemented with aspartate (10 mM), pyruvate (2 mM), or both metabolites. **(C-D)** Relative proliferation rate calculated from the growth curves shown in **(A-B)**. Confluence was normalized to cell number *t* = 0 following media supplementation and assessed over 72 h to determine proliferation rate. Statistical comparisons were performed using repeated-measures one-way ANOVA with Sidak’s multiple comparisons test. Values are mean ± SD. **p*<0.05, ***p*<0.01, ****p*<0.001, *****p*<0.0001, ns = not significant.


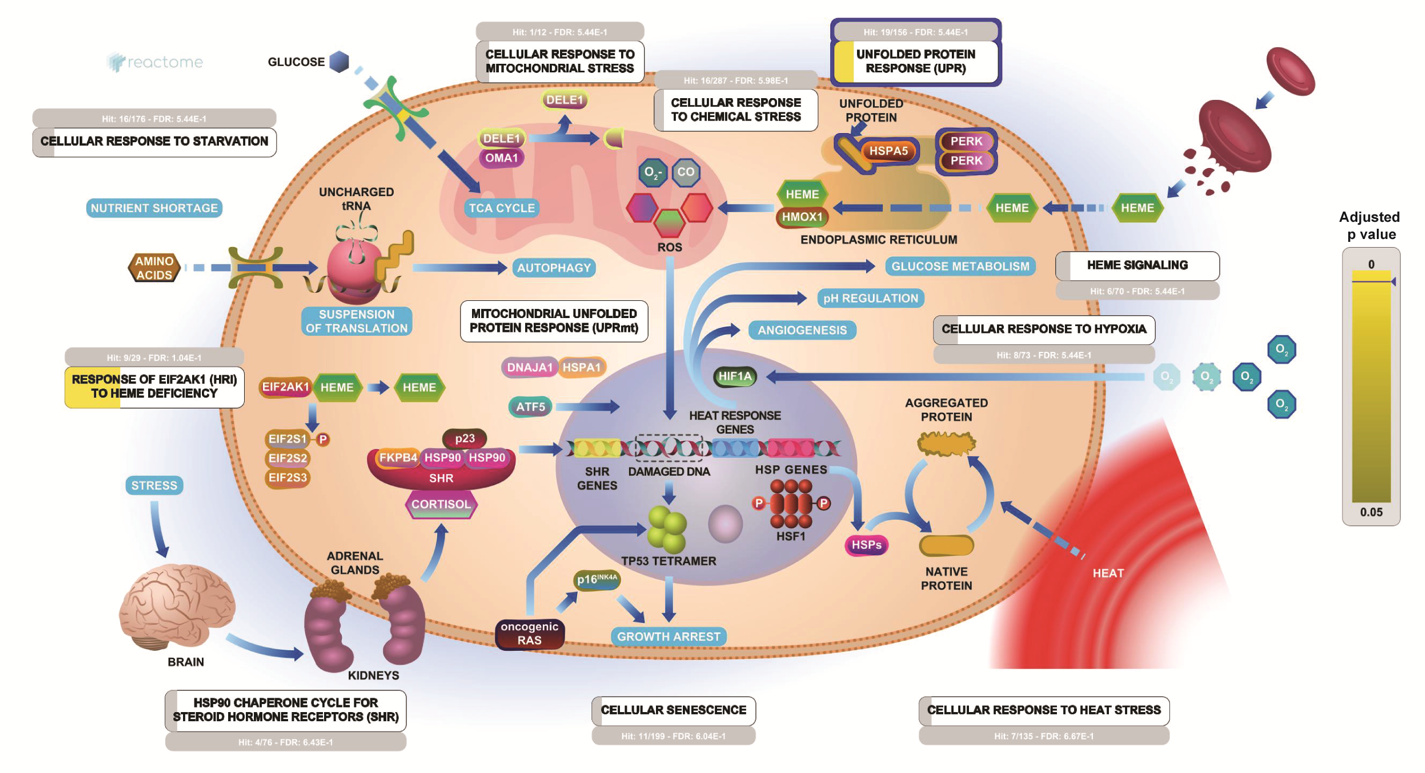


**Supplementary Figure 9. UPR signaling pathway activation was uncovered by unbiased bulk RNA sequencing analysis.** Full pathway diagram highlighting UPR signaling activation in Mito-Met_10_-treated MCW512 cells (1 μM for 6 h) as determined by pathway enrichment analysis using the Reactome Pathway Database (Reactome ID: 37941124) of DEG bulk RNA sequencing data. Figure created from the Reactome Pathway Database.

**
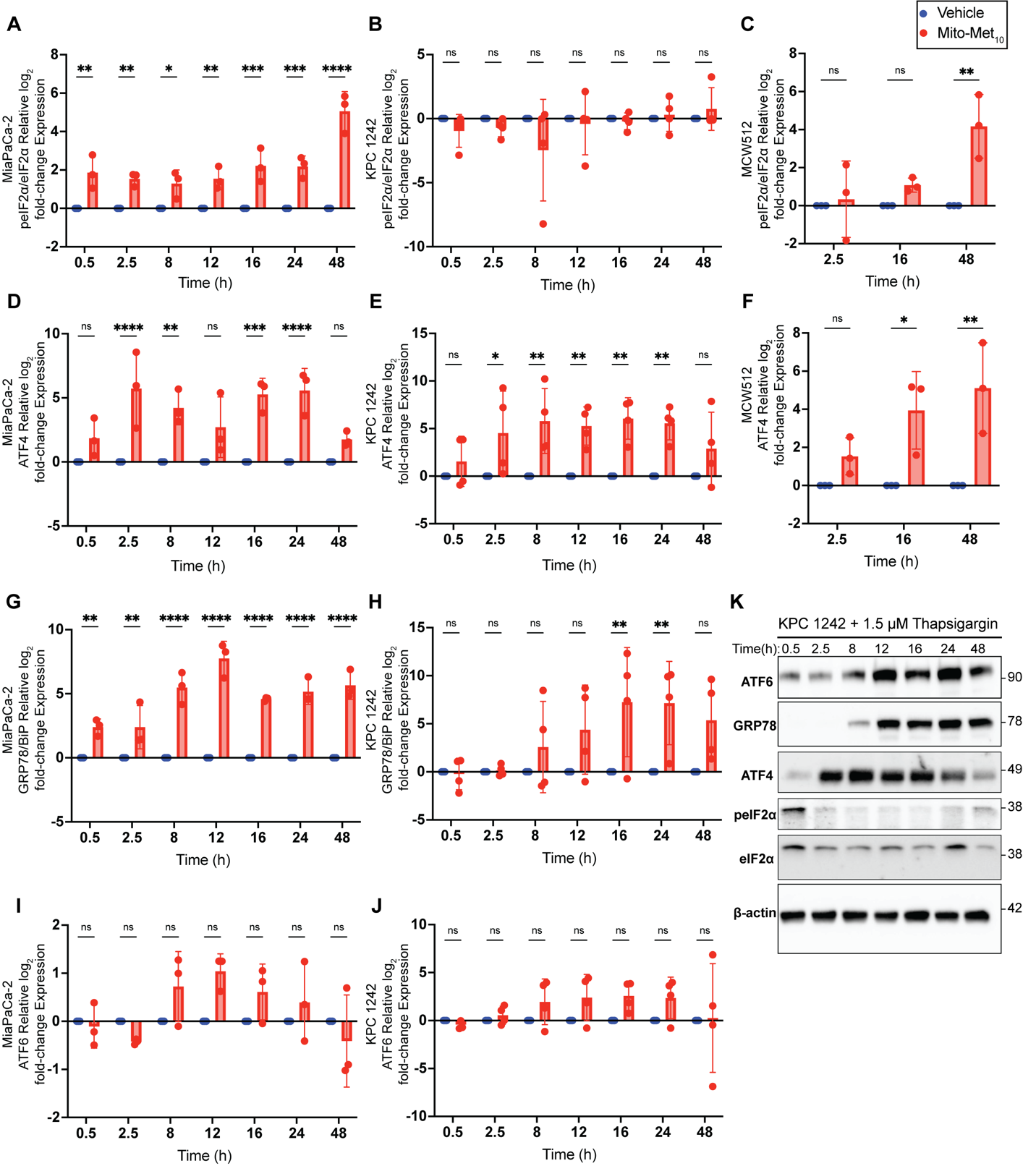
**

**Supplementary Figure 10. Mito-Met_10_ activates PERK-eIF2α-ATF4 signaling in human and murine PDAC cells.** MiaPaCa-2 (**A, D, I**), KPC 1242 (**B, E, H, J**), and **(C, F)** MCW512 cells were treated with vehicle (blue) or Mito-Met_10_ (*red*) (MiaPaCa-2: 0.3 μM; KPC 1242: 5 μM; MCW512: 1.5 μM) for the indicated time points. Densitometric quantification of **(A-C)** p-eIF2α/eIF2α ratio, (**D-F**) ATF4, **(G-H)** GRP78/BiP, and **(I-J)** ATF6 expression normalized to β-actin, quantified using Image Lab software. Statistical comparisons were performed relative to the vehicle at the corresponding time point within each cell line using two-way ANOVA with Sidak’s multiple comparisons test. **(K)** Representative immunoblots of ER stress pathway activation in KPC 1242 cells treated with thapsigargin (1.5 μM) for the indicated time points (0.5-48 h). Whole-cell lysates were probed for ATF6, GRP78/BiP, ATF4, phosphorylated eIF2α (p-eIF2α), eIF2α, and β-actin. Values are mean ± SD. **p*<0.05, ***p*<0.01, ****p*<0.001, *****p*<0.0001, ns = not significant.

**
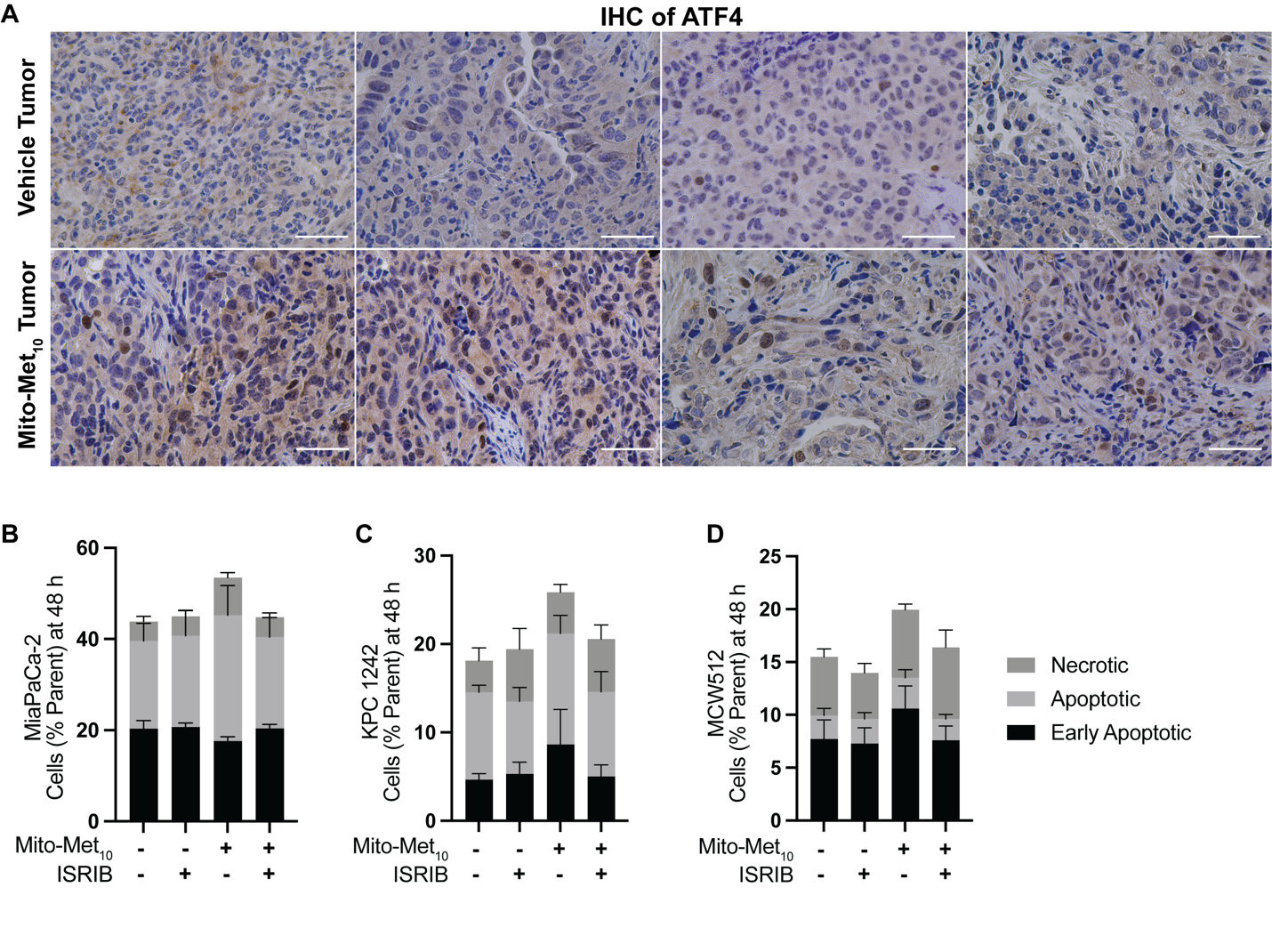
**

**Supplementary Figure 11. ISR inhibition attenuates Mito-Met_10_-induced apoptosis.**

**(A)** Representative immunohistochemical staining for ATF4 of each tumor section from vehicle-treated (n=4) and Mito-Met_10_-treated mice (2.5 mg/kg, n=4). Scale bars, 20 μm. **(B-D)** Quantification of necrotic, apoptotic, and early apoptotic cell populations in **(B)** MiaPaCa-2, **(C)** KPC 1242, and **(D)** MCW512 cells following treatment with vehicle or Mito-Met_10_ in the presence or absence of ISRIB (500 nM), corresponding to data in Figure 8G-I. Cells were treated with Mito-Met_10_ at cell line-specific concentrations (MiaPaCa-2: 0.4 µM; KPC 1242: 5 µM; MCW512: 1.5 µM) for 48 h. Viable (Annexin V^-^/PI^-^), early apoptotic (Annexin V^+^/PI^-^), late apoptotic (Annexin V^+^/PI^+^), and necrotic (Annexin V^-^/PI^+^) populations are indicated. Quadrant gates were defined using vehicle controls and applied uniformly across all conditions within each cell line. Values are mean ± SD. **p*<0.05, ***p*<0.01, ****p*<0.001, *****p*<0.0001

**Supplementary Table 1. Primer sequences.**

| **Gene** | **Species** | **Forward (5’ 🡪 3’)** | **Reverse (5’ 🡪 3’)** |
| --- | --- | --- | --- |
| DDIT3 | Human | TGA GTT GGC CAG GAC TTT AC | TTG GTC CCT GTA GCC ATT TC |
| Ddit3 | Mouse | CCA CCA CAC CTG AAA GCA GAA | AGG TGA AAG GCA GGG ACT CA |
| β-ACTIN | Human | CAC CAT TGG CAA TGA GCG GTT C | AGG TCT TTG CGG ATG TCC ACG |
| β-Actin | Mouse | CTC CCT GGA GAA GAG CTA TGA | CTA AGA AGG AAG GCT GGA AA |

**Supplementary Table 2. Mito-Met_10_ concentrations examined in each human and murine PDAC cell line analyzed.**

| **Cell Line** | **Concentration Range (μΜ)** |
| --- | --- |
| KPC 1242 | 0, 1, 2, 5, 10, 15, 20, 30, 40, 50, 60 |
| HPSC | 0, 5, 10, 20, 30, 40, 50, 60, 65, 70, 75 |
| MiaPaCa-2 | 0, 0.1, 0.2, 0.3, 0.35, 0.4, 0.45, 0.5, 0.55, 0.6, 0.65 |
| MCW512 | 0, 0.25, 0.5, 1, 3, 5, 10, 15, 20, 30, 60 |
