# Supplementary material for: Mitochondria-targeted metformin analogs activate the ER stress-unfolded protein response pathway to drive apoptosis in pancreatic cancer
Source: Cell Death Dis. 2026 May 22;17(1):643. doi: 10.1038/s41419-026-08859-y (PMC13372815; doi:10.1038/s41419-026-08859-y)

MiaPaCa-2 N=1

peIF2a

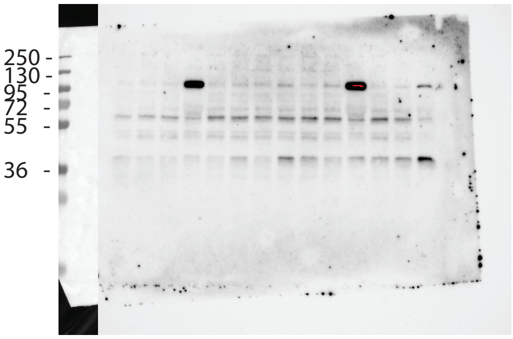

beta actin

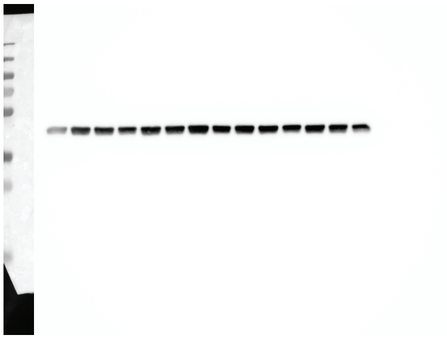

eIF2a

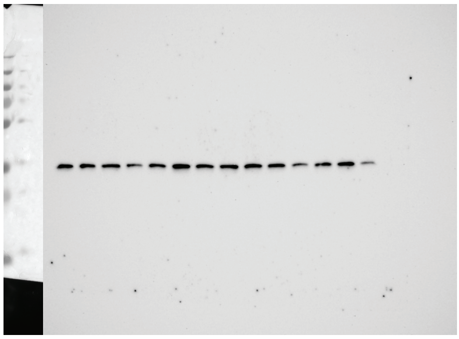

beta actin

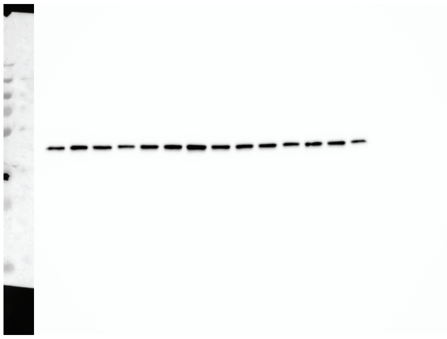

ATF4

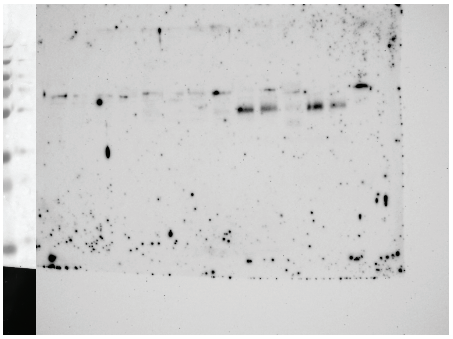

ATF6

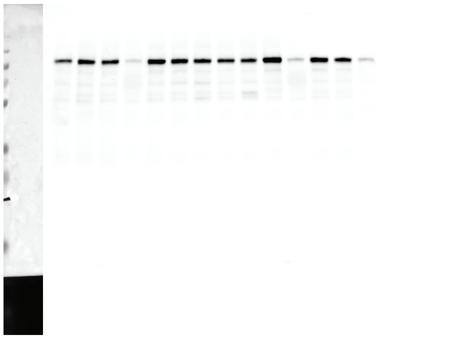

beta actin

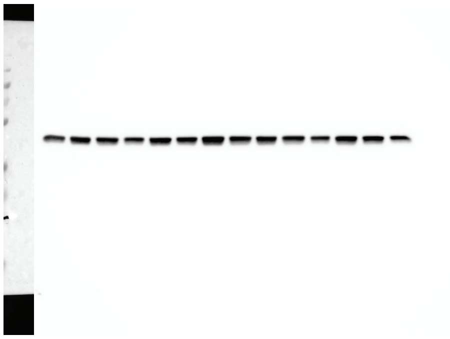

BiP

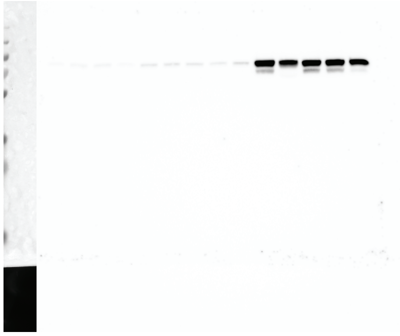

MiaPaCa-2 N=2

peIF2a

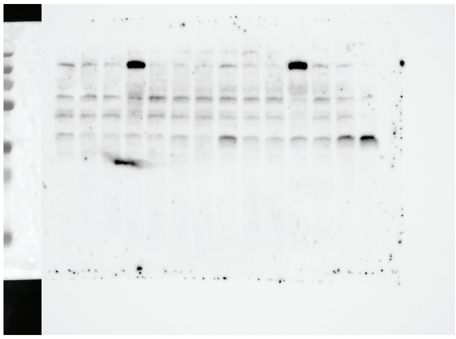

beta actin

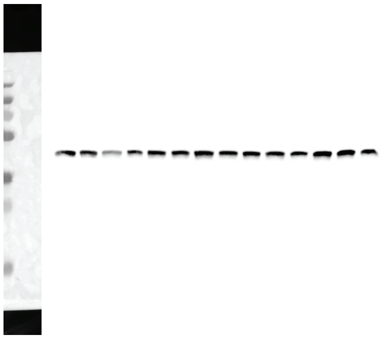

eIF2a

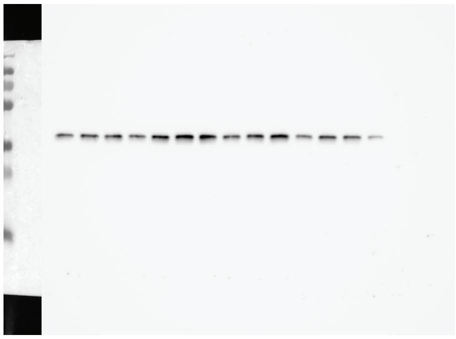

beta actin

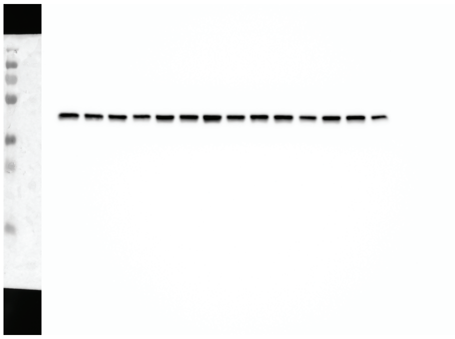

ATF4

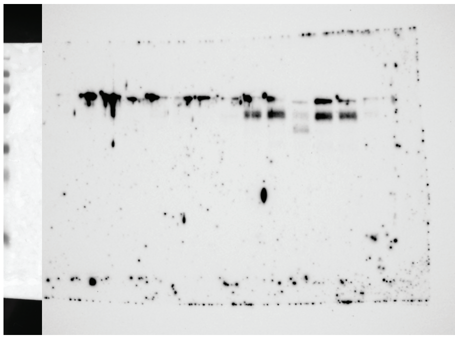

ATF6

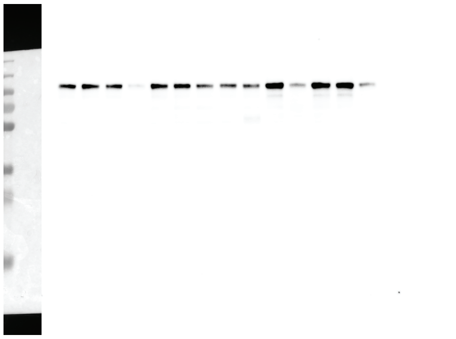

beta actin

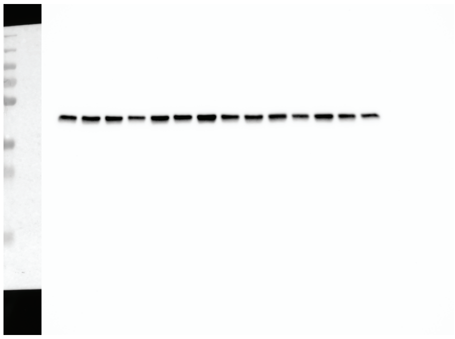

BiP

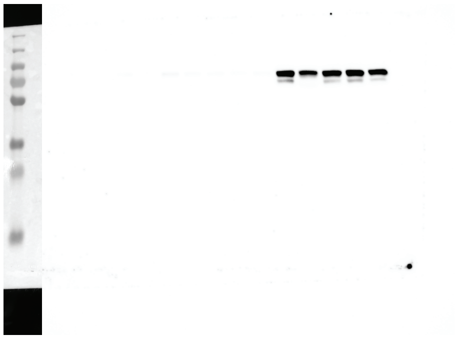

MiaPaCa-2 N=3

peIF2a

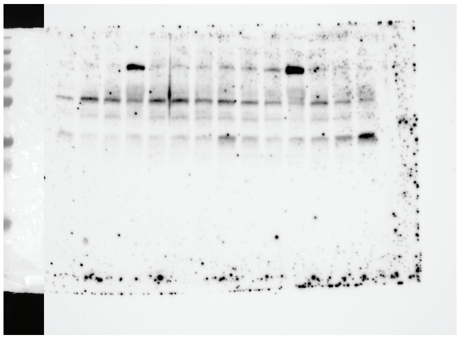

beta actin

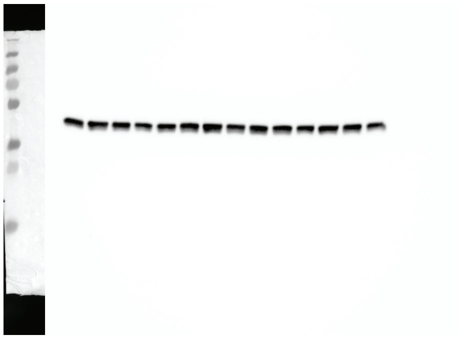

eIF2a

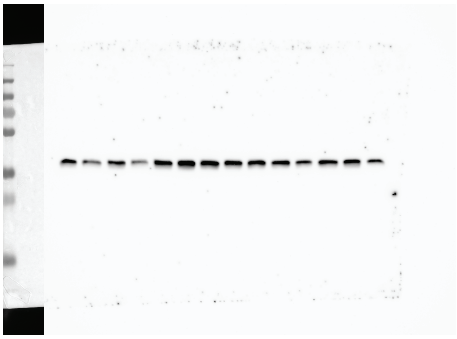

beta actin

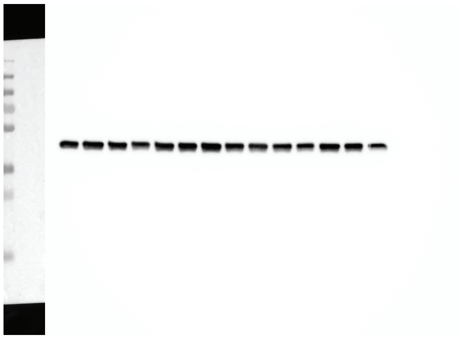

ATF4

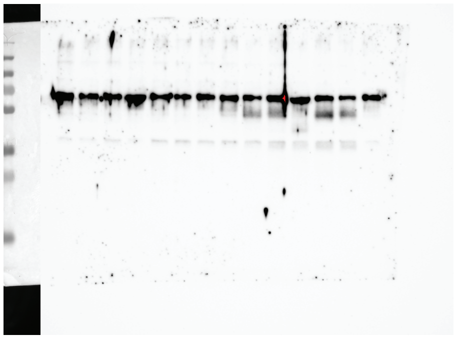

ATF6

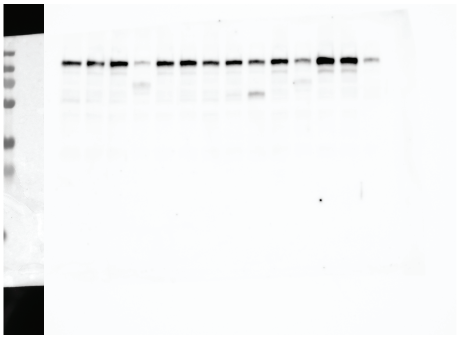

beta actin

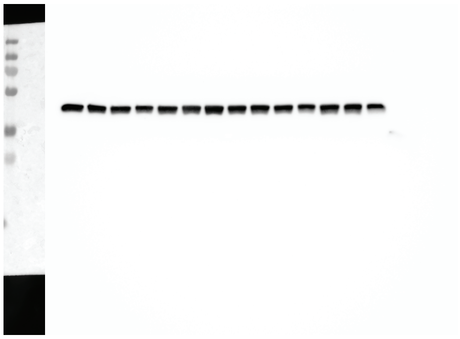

BiP

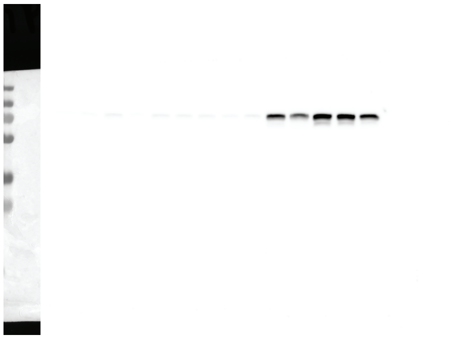

KPC 1242 N=1

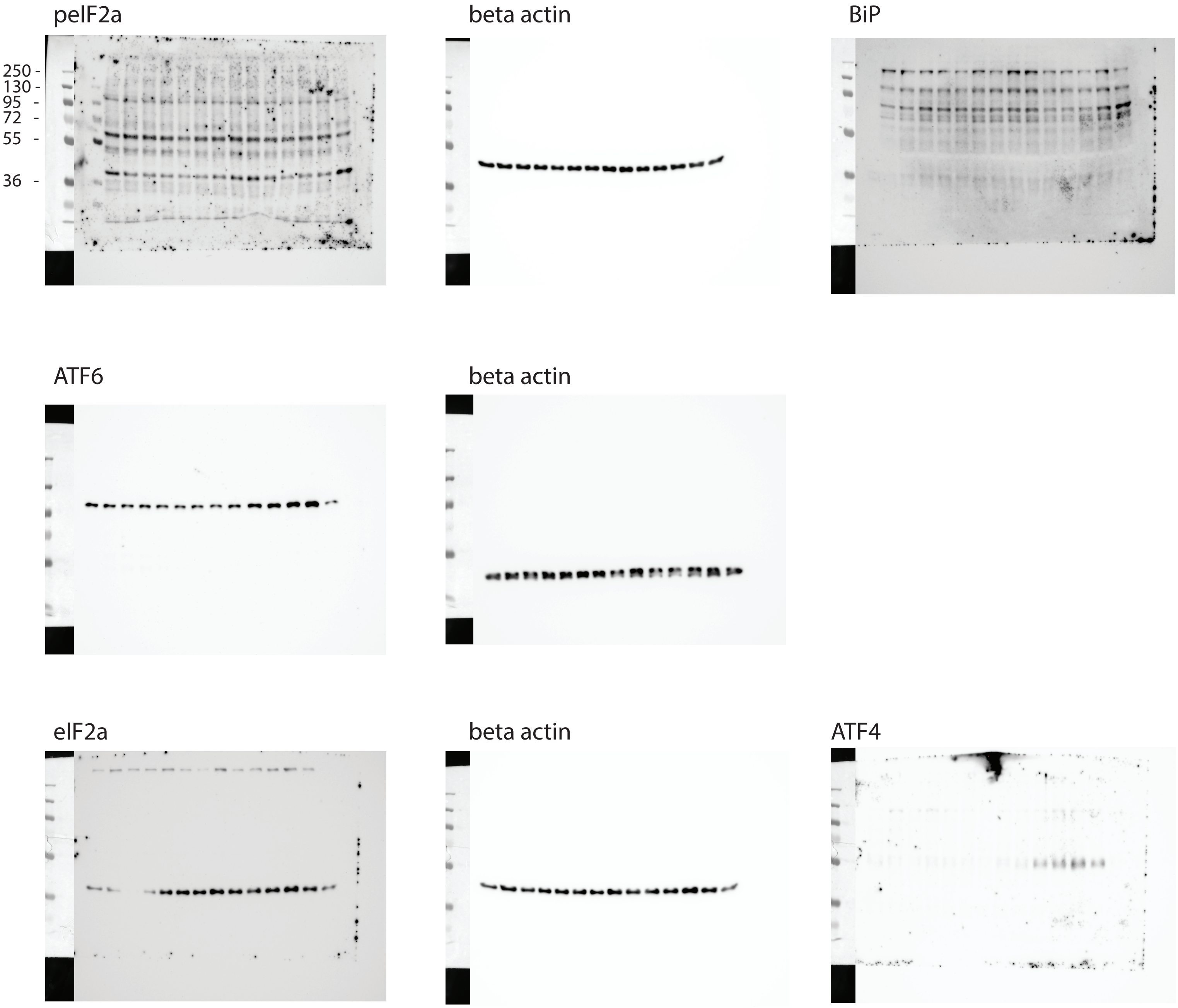
peIF2a
beta actin
BiP
250 -
130 -
95 -
72 -
55 -
36 -
ATF6
beta actin
eIF2a
beta actin
ATF4

KPC 1242 N=2

ATF4

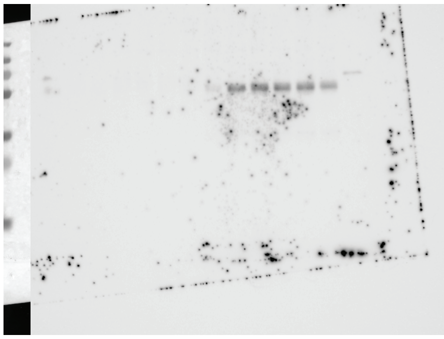

beta actin

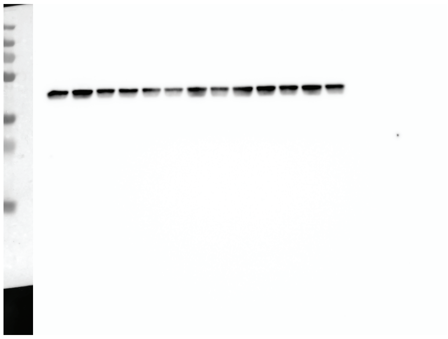

BiP

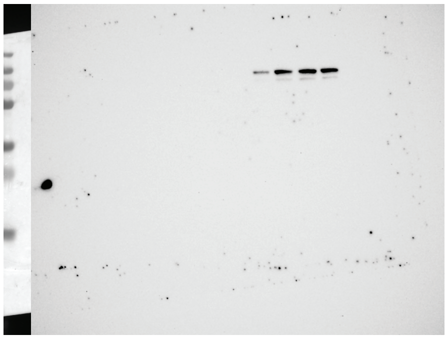

peIF2a

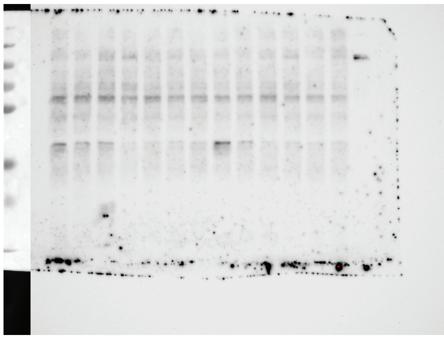

beta actin

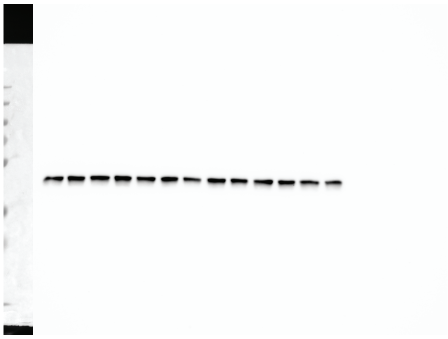

ATF6

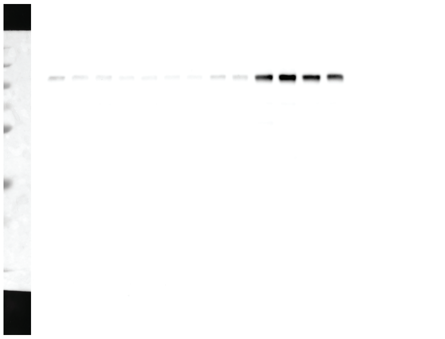

eIF2a

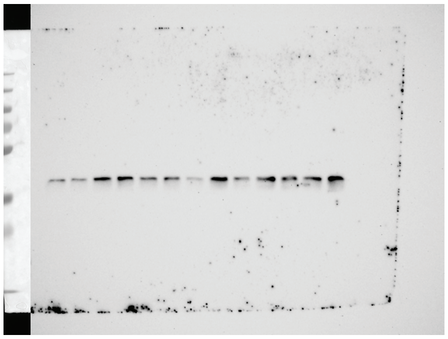

beta actin

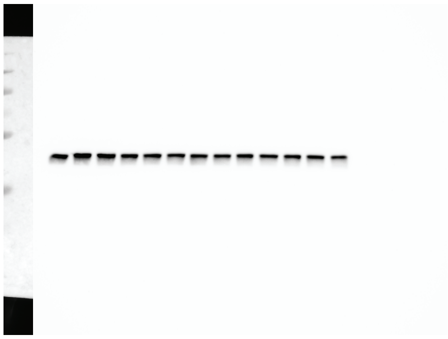

KPC 1242 N=3

ATF4

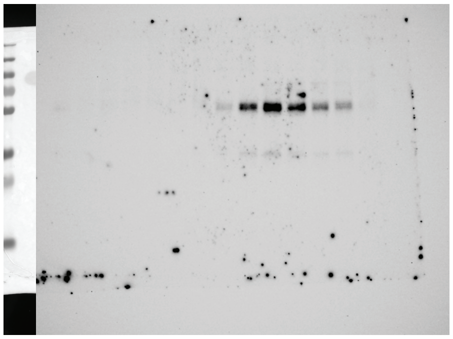

beta actin

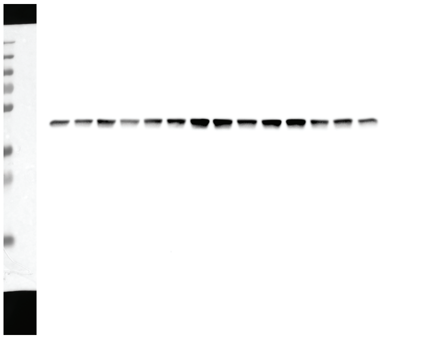

BiP

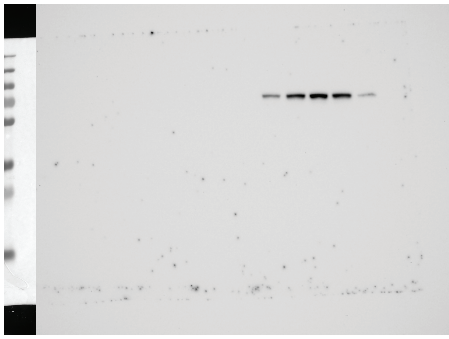

peIF2a

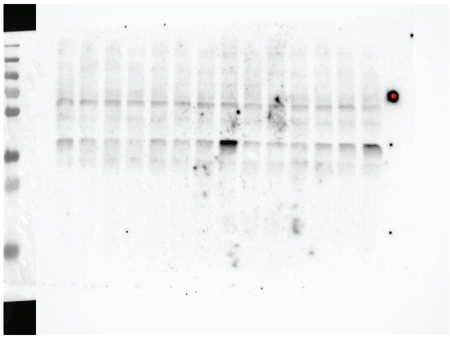

beta actin

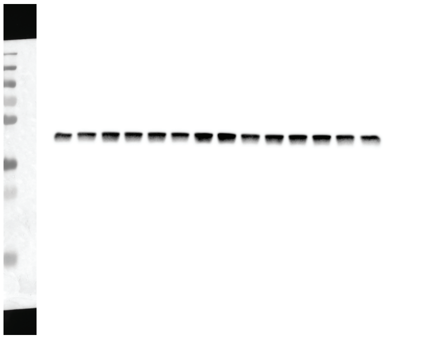

ATF6

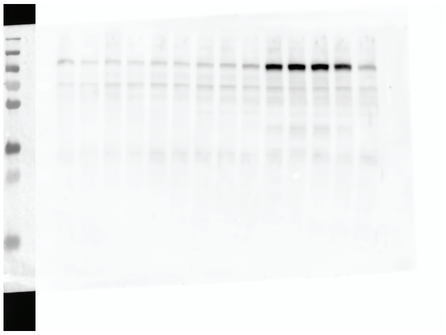

eIF2a

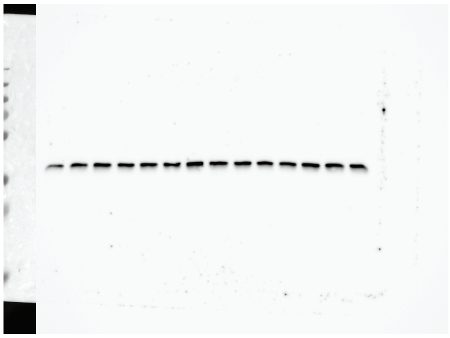

beta actin

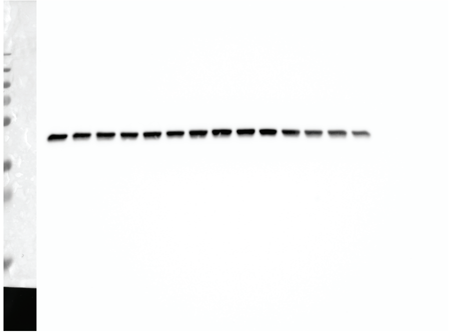

KPC 1242 N=4

ATF4

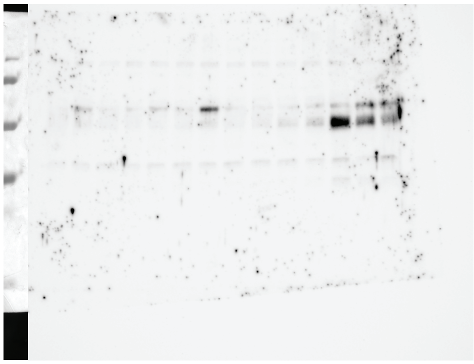

beta actin

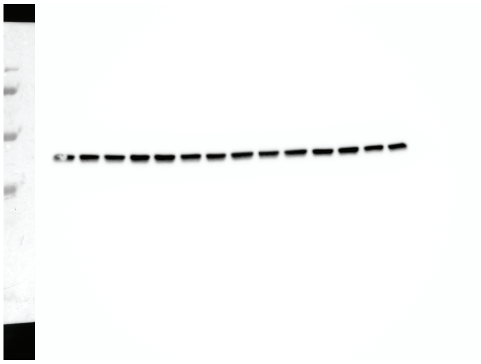

peIF2a

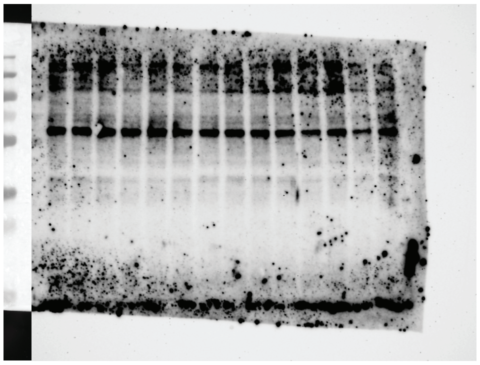

BiP

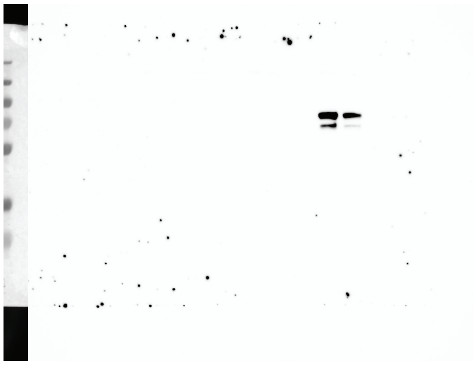

beta actin

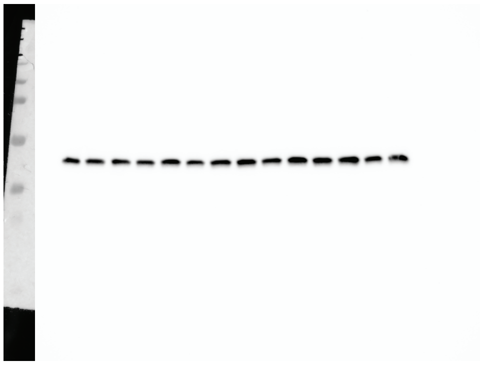

ATF6

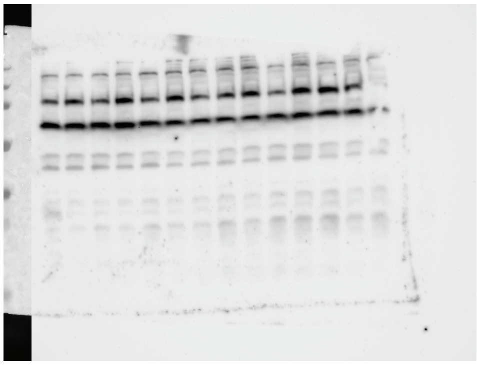

eIF2a

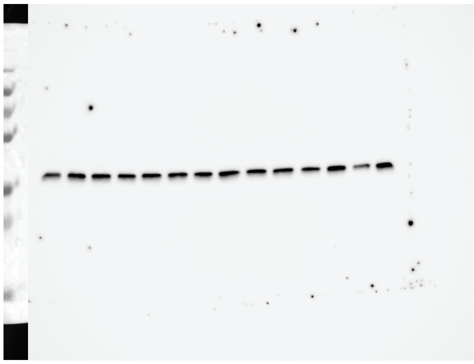

beta actin

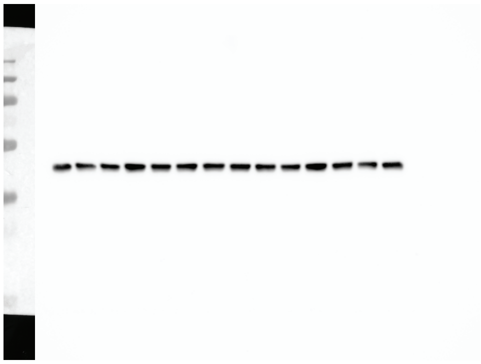

MCW512 N=1

ATF4

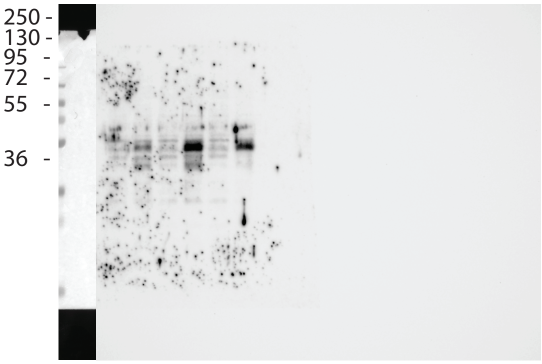

beta actin

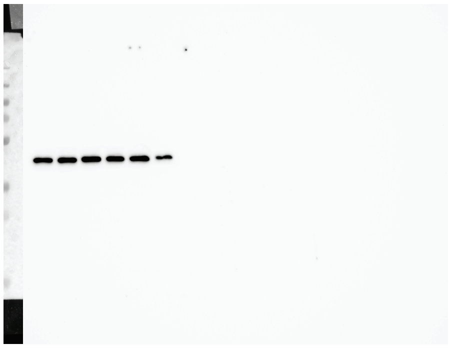

eIF2a

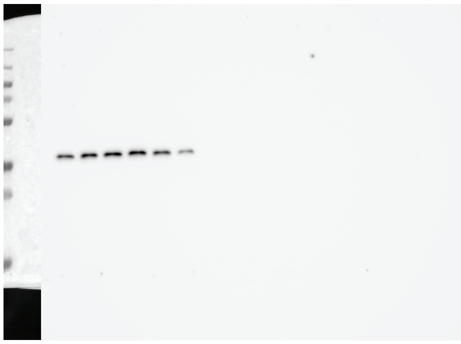

beta actin

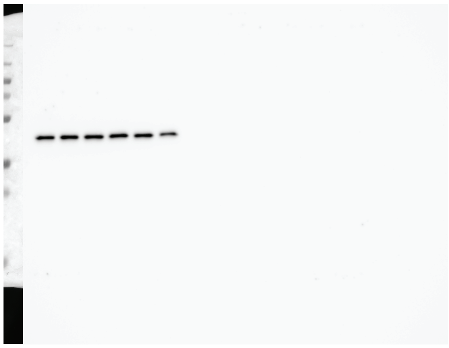

peIF2a

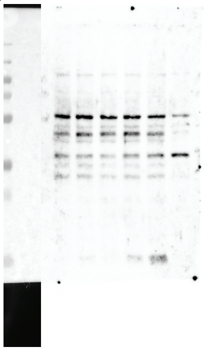

beta actin

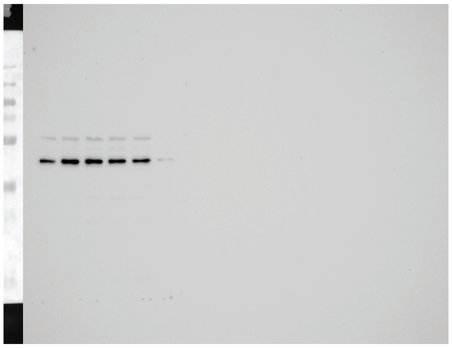

MCW512 N=2 MCW512 N=3 MCW512 N=2 MCW512 N=3

ATF4

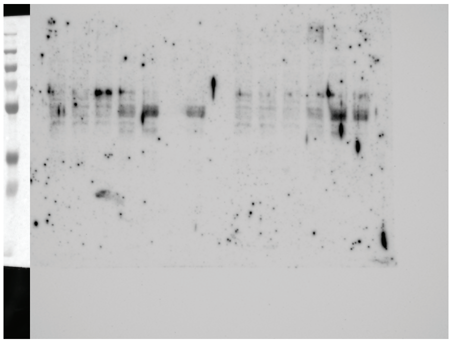

beta actin

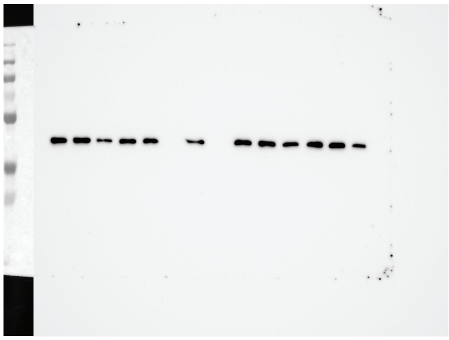

eIF2a

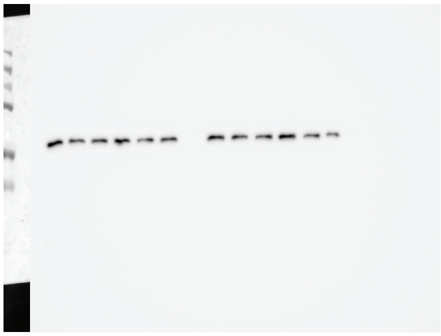

beta actin

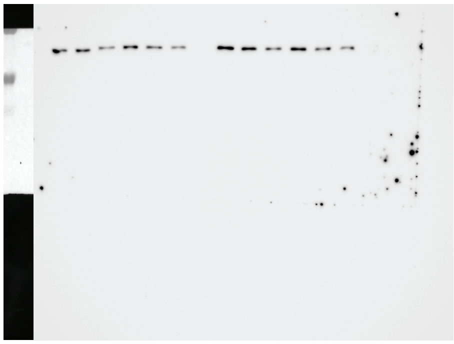

peIF2a

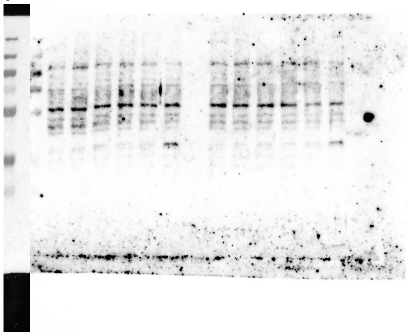

beta actin

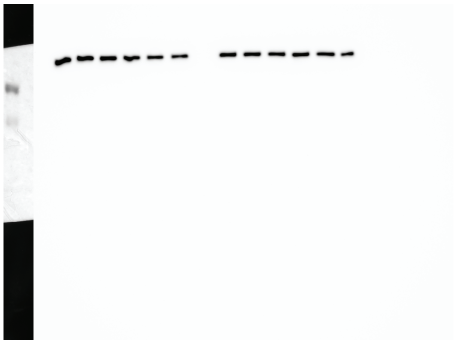

Supplement: Supplementary file 2 — Original Data [file 41419_2026_8859_MOESM2_ESM.pdf]
